# Field efficacy of *Bt* cotton containing events DAS-21023-5 × DAS-24236-5 × SYN-IR102-7 against lepidopteran pests and impact on the non-target arthropod community in Brazil

**DOI:** 10.1371/journal.pone.0251134

**Published:** 2021-05-04

**Authors:** Luiz H. Marques, Miles Lepping, Boris A. Castro, Antonio C. Santos, Jaedino Rossetto, Marcelo Z. Nunes, Oscar A. B. N. Silva, Valeria F. Moscardini, Verissimo G. M. de Sá, Timothy Nowatzki, Mark L. Dahmer, Pablo C. Gontijo

**Affiliations:** 1 Corteva Agriscience, Barueri, São Paulo, Brazil; 2 Corteva Agriscience, Indianapolis, Indiana, United States of America; 3 Corteva Agriscience, Johnston, Iowa, United States of America; 4 Instituto Federal Goiano, Campus Rio Verde, Rio Verde, Goiás, Brazil; Nigde Omer Halisdemir University, TURKEY

## Abstract

The efficacy and non-target arthropod effects of transgenic DAS-21023-5 × DAS-24236-5 × SYN-IR102-7 *Bt* cotton, expressing proteins Cry1Ac, Cry1F and Vip3Aa19, was examined through field trials in Brazil. Fifteen field efficacy experiments were conducted from 2014 through the 2020 growing season across six different states in Brazil to evaluate performance against key lepidopteran pests through artificial infestations of *Chrysodeixis includens* (Walker), *Spodoptera frugiperda* (J.E. Smith,1797), *Spodoptera cosmioides* (Walker, 1858) and *Chloridea virescens* (F., 1781), and natural infestations of *Alabama argillacea* (Hübner) and *S*. *frugiperda*. The impact of this *Bt* cotton technology on the non-target arthropod community in Brazilian cotton production systems was also assessed in a multi-site experiment. DAS-21023-5 × DAS-24236-5 × SYN-IR102-7 cotton significantly reduced the feeding damage caused by S. *frugiperda*, *S*. *cosmioides*, *C*. *includens*, *C*. *virescens* and *A*. *argillacea*, causing high levels of mortality (greater than 99%) to all target lepidopteran pests evaluated during vegetative and/or reproductive stages of crop development. Non-target arthropod community-level analyses confirmed no unintended effects on the arthropod groups monitored. These results demonstrate the value of transgenic *Bt* cotton containing event DAS-21023-5 × DAS-24236-5 × SYN-IR102-7 for consideration as part of an integrated approach for managing key lepidopteran pests in Brazilian cotton production systems.

## Introduction

The production of cotton (*Gossypium hirsutum* L.) plays an important role in the economy of many countries in tropical and subtropical regions of the world [1]. Arthropod pests have historically acted as major constraint on profitable cotton production and a limiting factor for the geographic expansion of the crop [1]. The cotton agroecosystem includes a wide range of arthropod species consisting of numerous key pests and hundreds of other species, such as beneficial species that prey upon or parasitize herbivorous pests [2, 3]. Several important cotton producing regions of the world can experience severe yield losses caused by insect pests that specialize their feeding on the cotton crop, such as boll weevil, *Anthonomous grandis* Boheman, and the pink bollworm, *Pectinophora gossypiella* (Saunders). Additionally, other pests common to cotton systems globally include a complex of heliothine species (Lepidoptera), as well as aphids, mirids, whiteflies (Hemiptera), thrips (Thysanoptera), and spider mites (Araneae). In particular, species in the genus *Spodoptera* have become increasingly important pests of cotton production in Brazil [4, 5]. Their impact and distribution depend largely on production systems and environmental conditions [1]. In recent years, S. *frugiperda* has expanded its geographic distribution and infested maize fields (*Zea mays* L.) in Africa [6, 7] and several Asian countries [8–10].

The history of cotton production exemplifies a reliance on a narrow range of pest management tactics and the subsequent challenges posed by pest adaptation to insecticides and the prevailing environment [1]. Cotton production in the United States relied heavily on chlorinated hydrocarbon, organophosphate, and carbamate insecticides in the 1960s and 1970s. The use of pyrethroid insecticides in the 1970s contributed to increased production and profitability but continued to intensify the use of chemical insecticides and resulted in the outbreak of secondary pest infestations [1].

Cotton breeding efforts have brought native insect antibiosis traits to many cotton varieties. A modern complement to pest-resistant cotton varieties expressing native traits and crop protection solutions includes cotton genetically transformed to express insecticidal proteins. The genes coding for insecticidal properties derived from the soil bacterium *Bacillus thuringiensis* (*Bt*) have been genetically engineered into several important crops (*Bt* crops). Brazil planted 51.3 million hectares of transgenic crops (including those that express insect and/or herbicide tolerance traits) in 2018, second only to the United States (75 million hectares) and followed by Argentina (23.9 million), Canada (12.7 million) and India (11.6 million). These five countries together planted an area of 191.7 million hectares of transgenic crops, representing 91% of the total global biotech crop area planted [11]. While the benefits of *Bt* crop technology vary by country and region, a reduction in insecticide use has been more noticeable in cotton production [12], thus promoting ecosystem services such as biological control.

*Bt* cotton was first launched commercially in 1996 in Australia, Mexico, and the United States. Commercial *Bt* cotton production in Brazil began in 2005 [13]. Since then, adoption of transgenic cotton reached 89.8% of the 1.44 million hectares of all cotton planted in Brazil by the end of the 2018–2019 growing season [14]. The area planted to transgenic cotton in Brazil during the 2018–2019 significantly increased by 48.3% compared with the previous season [14], which highlights the importance of *Bt* transgenic technology to manage key target pests under the tropical conditions of Brazilian agriculture.

Cotton production is heavily affected by a broad range of arthropod pests not targeted by currently available *Bt* cotton technologies. In Brazil, these pests include *A*. *grandis*, *Bemisia tabaci* (Gennadius), *Frankliniella schultzei* (Trybom), and *Aphis gossypii* Glover, etc., which may lead to significant impacts on yield if not controlled [15]. Thus, while *Bt* cotton represents an important component in the management of key lepidopteran pests, its compatibility with other pest management tactics is important to preserving populations of beneficial predators and parasitoids that help maintain other important pests below economically damaging levels. The season-long expression of *Bt* toxins in *Bt* crops is valuable to ensuring plant protection, with the concomitant expectation that non-target species might receive exposure in the agricultural landscape. Therefore, the assessment of environmental safety is a key component during the development process of transgenic crop technologies [13].

*Bt* cotton technology that expresses events DAS-21023-5 × DAS-24236-5 × SYN-IR102-7 was developed by Corteva Agriscience as a breeding stack of these three insect-protection events, which received approval by the Brazilian National Biosafety Technical Committee in 2018 [16]. The first commercial plantings in Brazil were conducted in 2019 under the trademarked name of WideStrike™ 3 Insect Protection (Corteva Agriscience, Wilmington, DE, United States). This technology is an advanced insect protection system that expresses the insecticidal delta-endotoxins Cry1Ac, derived from *B*. *thuringiensis* var. *kurstaki* strain HD73, expressed by event DAS-21023-5; Cry1F, derived from *B*. *thuringiensis* var. *aizawai* strain PS811, expressed by event DAS-24236-5, and the vegetative insecticidal protein Vip3Aa19, derived from *B*. *thuringiensis* strain AB88, expressed by event SYN-IR102-7.

As insect resistant traits are incorporated into seed products, the decision to plant *Bt* crops is made before planting, based on knowledge of key target lepidopteran pest infestations in areas where they are a perennial or an emerging threat [13]. While the ultimate decision to use *Bt* crops rests with the farmer, the continued offering of new traits, the stacking of commercially available traits, the choice of herbicide tolerance with or without insect resistant traits, etc., add complexity to decisions farmers must make [13]. Furthermore, Integrated Pest Management (IPM) considerations are extremely important for sustainable crop production. IPM theory encourages the adoption of selective tools that reduce populations of economic pests and offer additional benefits such as the protection of beneficial species including natural enemies and other non-target arthropods (NTAs) which can help manage secondary pest infestations [13, 17].

Problem formulation conducted as part of the environmental risk assessment for DAS-21023-5 × DAS-24236-5 × SYN-IR102-7 cotton considered the familiarity of the mode of action for Cry proteins [18–21], the narrow spectrum of activity for Cry proteins [22, 23], and demonstrated history of safe use for *Bt* crops [17, 24]. Previous laboratory studies using direct or indirect exposure test systems for the focal *Bt* proteins demonstrated no adverse effects on NTAs [25–32]. Continuing reviews [23, 33] and meta-analyses [13, 34, 35] of laboratory and field data support the safety of *Bt* proteins in each cropping system examined, including cotton, maize and soybean (*Glycine max* (L.) Merr.), in which they have been deployed. The primary differences in NTA populations observed between *Bt* crops and their conventional (non-*Bt*) counterparts (in the absence of insecticide applications) have been attributed to reductions in lepidopteran pest abundance and/or prey quality, which may simplify the dynamic of the system. Based on the existing data supporting the safety of *Bt* proteins broadly, and for Cry1Ac, Cry1F and Vip3A specifically, the problem formulation step for DAS-21023-5 × DAS-24236-5 × SYN-IR102-7 concluded that additional testing was not required to refine the risk assessment. Nevertheless, to supplement existing data and to meet regulatory requirements, NTA field trials were incorporated into the risk assessment.

Therefore, the objectives of this study were to evaluate the field efficacy of the *Bt* cotton technology expressing events DAS-21023-5 × DAS-24236-5 × SYN-IR102-7 on a complex of key lepidopteran pests in Brazil and to assess its impact on the non-target arthropod community associated with Brazilian cotton production systems.

## Materials and methods

### Control of lepidopteran pests

Fifteen field experiments were conducted from 2014 through the 2020 growing season across six different states in Brazil (Table 1). Field sites were located across central Brazil in areas of commercial cotton production that represented distinct agronomic practices and environmental conditions typical of cotton producing areas. Treatments included: 1) A *Bt* cotton variety containing events DAS-21023-5 × DAS-24236-5 × SYN-IR102-7 (WideStrike™ 3 Insect Protection, Corteva Agriscience, Wilmington, DE) expressing Cry1Ac, Cry1F and Vip3Aa19 transgenic proteins, and 2) A non-*Bt* isoline cotton variety containing the same genotypic background and belonging to the same maturity group as the *Bt* cotton variety. The *Bt* cotton variety used was PHY440WS3 (Mid-full Maturity, Mycogen® seeds) in all treatments until 2018. In 2019 and 2020 field trials, the varieties used were an experimental variety from TMG (Tropical Melhoramento & Genética S.A–Cambé, Paraná, Brazil). Each field trial consisted of four replications for each treatment arranged in a randomized complete block (RCB) design. Plot size varied among locations from five (5) to eight (8) m in length and five or seven rows wide. Row spacing in all locations varied from 50 to 76 cm.

**Table 1:** Trial locations, target pests and infestation type for each study year (2014 to 2020) in Brazil.

Trial Location (city, state)	Geographic coordinates	Year	Insect target
***Efficacy trials***			
Conchal, SP	22°24′09.30′′ S 47°07′14.60′′ W	2014	*C*. *includens*, *C*. *virescens*, *S*. *frugiperda*
Indianópolis, MG	18°57′29.70′′ S 47°51′21.10′′ W	2014	*A*. argillacea*, *C*. *includens*, *C*. *virescens*, *S*. *cosmioides*, *S*. *frugiperda*
	18°57′24.69′′ S 47°51′12.09′′ W	2016	*C*. *includens*, *C*. *virescens*, *S*. *cosmioides*, *S*. *frugiperda*
	18°57′08.60′′ S 47°51′11.80′′ W	2016	*C*. *includens*, *C*. *virescens*, *S*. *cosmioides*, *S*. *frugiperda*
	24°47′14.20′′ S 49°53′02.00′′ W	2018	*C*. *includens*, *C*. *virescens*, *S*. *cosmioides*, *S*. *frugiperda*
Montividiu, GO	17°22′33.15′′ S 51°23′46.36′′ W	2014	*A*. argillacea*, *C*. *includens*, *C*. *virescens*, *S*. *frugiperda*
Palotina, PR	24°21’43.00" S 53°45’23.70" W	2016	*A*. argillacea*, *C*. *includens*, *C*. *virescens*, *S*. *cosmioides*, *S*. *frugiperda*
Rio Verde, GO	17°45′02.20″ S 51°02′18.30″ W	2016	*C*. *includens*, *S*. *frugiperda*
	17°45′24.40″ S 51°02′04.90″ W	2017	*C*. *includens*, *C*. *virescens*, *S*. *cosmioides*, *S*. *frugiperda*
	17°45′18.20″ S 51°02′08.10″ W	2018	*C*. *includens*, *C*. *virescens*, *S*. *cosmioides*, *S*. *frugiperda*
São Desidério, BA	12°40′10.00″ S 45°57′56.00″ W	2019	*S*. frugiperda*
	12°40′11.00″ S 45°57′55.00″ W	2019	*S*. frugiperda*
	12°40′33.00″ S 45°58′02.00″ W	2020	*S*. frugiperda*
Sorriso, MT	12°27′34.27” S 55°49′41.12” W	2017	*C*. *includens*, *C*. *virescens*, *S*. *cosmioides*, *S*. *frugiperda*
Uberlândia, MG	18°54’08.09" S 48°10’02.24" W	2019	*A*. argillacea*
***Non-target Arthropod trials***			
Conchal, SP	22°24′09.28′′ S 47°07′14.59′′ W	2014	-
Indianópolis, MG	18°57′29.92′′ S 47°51′11.02′′ W	2015	-
Montividiu, GO	17°22′40.40′′ S 51°23′39.58′′ W	2014	-

*Natural infestation; otherwise artificial infestation.

### Artificial insect pest infestations

All treatments were evaluated against *Spodoptera cosmioides* (Walker, 1858), *Chrysodeixis includens* (Walker), *Spodoptera frugiperda* (J.E. Smith,1797) and *Chloridea virescens* (F., 1781) (Lepidoptera: Noctuidae) utilizing artificial infestations at all locations to ensure uniform pest pressure across experimental plots (Table 1). Insects were obtained from laboratory-reared colonies maintained by Corteva Agriscience (Mogi Mirim Research Center, Mogi Mirim, São Paulo State, Brazil). Laboratory colonies were maintained on artificial insect diets following the recommendations from Greene et al. [36]. Colony vigor was maintained by introducing new field-collected larvae every year from cotton, maize and soybean fields that also serve as hosts for these pests. Artificial infestations were conducted at four different phenological cotton growth stages defined by the BBCH Scale [37]. The phenological stages for infestation were chosen based on the time at which infestations normally occur for each species and minimizing overlap with natural infestations of boll weevil, *A*. *grandis*, during our trials. During the vegetative stages, artificial infestations of *C*. *includens* and *S*. *cosmioides* were conducted at GS1: 15 (cotton with 5–6 leaves) and GS1: 15+, 10–12 days later. During the reproductive stages, artificial infestations of *S*. *cosmioides*, *S*. *frugiperda* and *C*. *virescens* were conducted at GS6: 65, beginning of flowering (“mid bloom”), followed by a second infestation at GS6: 65+, 10–12 days later. For each plot, ten plants were randomly selected and each one was infested with ten first instars (L1). Larvae were placed on the growing points of the selected plants, and then covered immediately after with mesh cages (150 cm long × 50 cm wide × 150 cm high) to limit larval escape and to avoid mortality caused by natural enemies. Field evaluations for *C*. *includens* and *S*. *cosmioides* included percent visual defoliation (0–100%) and the number of live larvae, both recorded 10 days after infestation (DAI). Evaluations for *S*. *cosmioides*, *S*. *frugiperda* and *C*. *virescens* infested during reproductive stages consisted of recording the total number of cotton squares on ten infested plants, the percentage of damaged squares, and the number of live larvae still present.

### Natural infestation

The efficacy of *Bt* cotton technology with events DAS-21023-5 × DAS-24236-5 × SYN-IR102-7 was evaluated against natural infestations of *Alabama argillacea* (Hübner) (Lepidoptera: Noctuidae) and *S*. *frugiperda* at a subset of locations (Table 1). Where *A*. *argillacea* infestations naturally occurred, evaluation included percent defoliation (0–100%) estimated visually by observing the amount of defoliation in the entire plot. For *S*. *frugiperda*, 25 cotton squares per plot were randomly selected from five plants from the two center rows per plot. The visual evaluations included counting the total number of damaged squares, the percentage of damaged squares and the number of live larvae found after manually inspecting the reproductive plant parts. Plot evaluations were performed weekly. The data presented in this paper represent the sampling dates when peak defoliation and number of damaged squares were recorded for the non-*Bt* treatment at each location.

### Effects on non-target arthropods

Field trials were conducted in Conchal, São Paulo State; Indianópolis, Minas Gerais State; and Montividiu, Goiás State, Brazil during the 2014/2015 cropping season (Table 1) to assess the impact of treatments on non-target arthropods (NTAs). Treatments included: 1) A *Bt* cotton variety containing events DAS-21023-5 × DAS-24236-5 × SYN-IR102-7, and 2) A non-*Bt* cotton isoline containing the same genotypic background and belonging to the same maturity group as the *Bt* cotton variety. Plot size at each site was 17 rows wide (50-cm row centers) and 20 m in length. The *Bt* cotton variety, PHY440WS3 (Mid-full Maturity, Mycogen® seeds) was used in all treatments. Each site included four replications per treatment arranged in a randomized complete block (RCB) design. Arthropods were collected using the following sampling methods: beat cloth, yellow sticky card trapping, pitfall trapping, and Berlese-Tullgren funnel extraction of NTAs from samples of litter and/or soil. Sampling with each method was conducted at GS1: 13, GS5: 51; GS6: 65; GS7:75; and GS9: 95 cotton growth stages [37].

### Foliar-dwelling non-target arthropod sampling

A white cloth (1 m long × 0.5 m wide) was used to collect foliar-dwelling arthropods at sampling points that included two rows of plants along a one-meter length of row. Collections included four sampling points per plot during each cotton growth stage, except GS1: 13, as plants were too small for beat cloth sampling. During GS1: 13, the plants within each sampling point were visually inspected and the arthropods were counted and identified. For all other samples, the beat cloth was placed on the ground between cotton plant rows, and the plants on either side were bent over the cloth and shaken vigorously. Arthropods that were dislodged from foliage onto the cloth were counted and recorded. The arthropod fauna collected at each point were identified to the species and morphospecies level in the field when possible. The unidentified species were placed in labeled containers containing 70% ethylic alcohol and transported to the laboratory for further identification.

### Aerial arthropod sampling

Yellow sticky cards were deployed to estimate relative numbers of small flying insects and other arthropods active in the cotton canopy. During each crop stage monitored, six yellow sticky cards were placed at equal distances apart within the central sampling area of each plot. Cards were supported on sticks or cane poles at the apex canopy height during GS1: 13, alongside the first inflorescences during GS5: 51; alongside the most developed flowers during GS6: 65; at cotton boll height during GS7: 75 and GS8: 85; and within the middle third of the plant canopy in GS9: 95. Each exposed card was collected and placed inside a resealable plastic bag with the sticky side adhered smoothly to the transparent side of the bag. Bags were marked with plot identification codes and brought to the laboratory, where captured arthropods were identified through the bag wall under a stereomicroscope to taxonomic order or family.

### Surface-dwelling arthropod sampling

Pitfall trapping was used to monitor surface-dwelling arthropods during cotton stages GS1: 13, GS5: 51; GS6: 65; GS7: 75; and GS9: 95. During each sampling period, two pitfall traps were placed near the center of each plot to reduce edge effects. Traps were spaced 4 m apart within a single row interspace. Each trap consisted of a plastic outer cup (8 cm in diameter x 14 cm depth) buried in the soil with the upper rim positioned at ground level. A galvanized tripod shield was placed over each cup with a gap of 2–3 cm between the rim of the cup and the shield to protect against rain and to reduce debris contamination. The traps were filled with a mixture of water, formaldehyde (10%) and a few drops of detergent soap and left in the field for three days during each sampling period. The contents were sieved using a fine (0.5 mm) mesh sieve [38, 39] labeled and preserved in 70% ethanol. Arthropods were identified in the laboratory. The most representative arthropods were identified to the family level or higher taxonomic resolution. Sampled arthropods were also assigned to an ecological function based on family habits, or subfamily habits for taxonomic groups with multiple feeding habits [40].

To collect micro-arthropods from the soil, two soil blocks were taken randomly from each plot. Five subsamples were collected per sample. Each sub-sample consisted of both soil and litter and were collected using a manual digger to a depth of 5 cm below ground level. The total amount of soil and litter per sample was approximately 1.5 L. Samples were collected during the morning period to avoid collection of waterlogged samples that could occur during other times of day following rain events. Soil samples were transported to the laboratory in plastic containers to limit soil disruption. Containers were transported in rigid polystyrene foam boxes with ice packs and subsequently stored at ~22°C at each site for a maximum of 48 hours prior to delivery to the extraction laboratory. Samples were then placed in Berlese-Tullgren funnels for arthropod extraction over a 72-hour period. As a source of heat and desiccation, 60-watt incandescent light bulbs were placed above the samples during the extraction period. Extracted arthropods were deposited into collection vessels containing 70% ethanol as a preservative. Specimens were observed under a stereomicroscope for identification.

### Agronomic practices

All efficacy and NTA trials conducted from 2014–2018 followed strict adherence to Brazilian regulatory requirements and were therefore conducted at accredited and certified field research stations operated by Corteva Agriscience or SGS Company. Field trials conducted between 2014 and 2018 were performed under regulated permits approved by the Comissão Técnica Nacional de Biossegurança (CTNBio). Studies performed in 2019 and 2020 were conducted at commercial farms following the regulatory approval of DAS-21023-5 × DAS-24236-5 × SYN-IR102-7 cotton for field planting in Brazil. Conventional tillage was applied at each site, except in the efficacy trial conducted in Palotina, PR, which utilized no-tillage practices. The soil type at all sites was loam. Plots were seeded at a density of ten seeds per linear meter. Standard agronomic practices were used for fertilization, irrigation, disease, and weed management. Crop management practices during the study excluded the use of sprayed insecticides or miticides.

### Statistical analyses

#### Control of lepidopteran pests

Efficacy data on lepidopteran pests were subjected to a combined, cross-trial analysis using a linear mixed model where statistical significance was determined using an F-test (PROC MIXED; [41]) with α = 0.05. Prior to the combined analysis, each trial was analyzed individually and the mean square error of the residual (MSE) was used to evaluate the homogeneity of the variance error. Only trials that showed a ratio between the largest and smallest MSE ≤ 7 were included in the combined analysis [42]. This procedure ensured that variance across trials was sufficiently homogeneous to avoid bias caused by differences among trials (sites and years). To improve the distribution of data towards the assumption of normality, percentages were log (x +1) transformed, while data on number of larvae were transformed using square root (x+1). Non-transformed data are presented in all figures.

#### Effects on non-target arthropods

The potential impact of *Bt* cotton on the community of monitored NTAs associated with cotton fields was investigated using the Principal Response Curve (PRC) method. For this, the abundance of NTAs collected were subjected to Redundancy Analysis (RDA) and the significance of the first canonical axis (hereafter, *first axis*) was tested by Monte-Carlo permutation test (999 permutations) [43]. Before RDA, the abundance of the NTAs was log (x+1) transformed to reduce the effect of highly abundant taxa. Additionally, only taxa that exhibited a collection frequency ≥ 10% were included in the analysis. The data matrix distribution model was also examined using Detrended Correspondence Analysis (DCA) to ensure that the RDA linear method was appropriate, where a gradient length of < 4.0 was used as the criterion for acceptability [44] (S1 Table). After RDA, PRC diagrams based on variation captured in the first axis were constructed for each sampling method (beat cloth, sticky card trapping, pitfall trapping and Berlese-Tullgren funnel extraction) by site (Conchal, Indianópolis and Montividiu). The taxon weight (*bk*) for each NTA estimated by the analysis can be interpreted as the affinity of the taxon with the principal response curve (*Cdt*), where positive weights indicate that taxon abundances follow the PRC curve trend and negative weights follow an opposite trend [43]. All analyses were performed using CANOCO 4.5 software [45] with α = 0.05. According with Van den Brink and Ter Braak [43], when the first axis was statistically significant, the abundance of NTAs that most contributed to the community response in the PRC (taxon weights greater than 0.5 or less than -0.5, *hereafter > |0*.*5|*) was subjected to two-way repeated-measures ANOVA (two-way RM-ANOVA). This analysis was performed to test for interaction between the effects of the fixed factors, cotton technology (*Bt* or non-*Bt*) and sampling time (cotton growth stage) using PROC MIXED at α = 0.05 [41]. Blocked replicates were considered a random factor. When necessary, abundance was log transformed prior to analysis; non-transformed means are presented.

## Results

### Control of lepidopteran pests

A cross-trial analysis (sites and years) examining the efficacy of DAS-21023-5 × DAS-24236-5 × SYN-IR102-7 *Bt* cotton, expressing proteins Cry1Ac, Cry1F and Vip3Aa19, on key lepidopteran pests under artificial and natural infestation scenarios presented consistent results for vegetative and reproductive cotton growth stages (Table 2).

**Table 2:** Results of linear mixed model analyses using an *F*-test (α = 0.05) to compare efficacy of *Bt* cotton technology with events DAS-21023-5 × DAS-24236-5 × SYN-IR102-7 and non-*Bt* cotton against lepidopteran pests at different cotton growth stages.

Trial type	Target insect pest	Cotton growth stage^1^	Parameter	Degrees of freedom		Stat. values		Figure
				Num	Den	F	*P*	
Artificial infestation	*Chrysodexis includens*	GS1: 15	Defoliation	1	6	27.04	0.0020	1A
			No. live larvae	1	6	28.85	0.0017	1B
		GS1: 15+	Defoliation	1	9.72	49.76	0.0001	1C
			No. live larvae	1	11.5	34.37	0.0001	1D
	*Chloridea virescens*	GS6: 65	Square attacked	1	6	26.09	0.0022	1E
			No. live larvae	1	10	31.88	0.0002	1F
		GS6: 65+	Square attacked	1	5	30.54	0.0027	1G
			No. live larvae	1	4	11.55	0.0273	1H
	*Spodoptera frugiperda*	GS6: 65	Square attacked	1	7.93	42.34	0.0002	1I
			No. live larvae	1	9.99	7.51	0.0208	1J
		GS6: 65+	Square attacked	1	5	33.20	0.0022	1K
			No. live larvae	1	38	75.40	0.0001	1L
	*Spodoptera cosmioides*	GS1: 15	Defoliation	1	3	25.33	0.0151	2A
			No. live larvae	1	10	19.38	0.0013	2B
		GS1: 15+	Defoliation	1	1	231.66	0.0291	2C
			No. live larvae	1	2	24.18	0.0438	2D
		GS6: 65	Square attacked	1	4	16.84	0.0148	2E
			No. live larvae	1	4	20.87	0.0103	2F
		GS6: 65+	Square attacked	1	4	32.88	0.0046	2G
			No. live larvae	1	3	19.78	0.0422	2H
Natural infestation	*Alabama argillacea*^2^	Vegetative and Reproductive	Defoliation	-				3A
	*Spodoptera frugiperda*	Reproductive	Square attacked	1	3.16	218.92	0.0005	3B
			No. live larvae	1	2	40.21	0.0240	3C

^1^Phenological growth stages of the cotton plant classified according to Munger et al. (1998).

^2^Statistical test not applied due to a numerical difference sufficiently large enough to declare a difference between treatments means.

#### Artificial infestations

**Fig 1:**
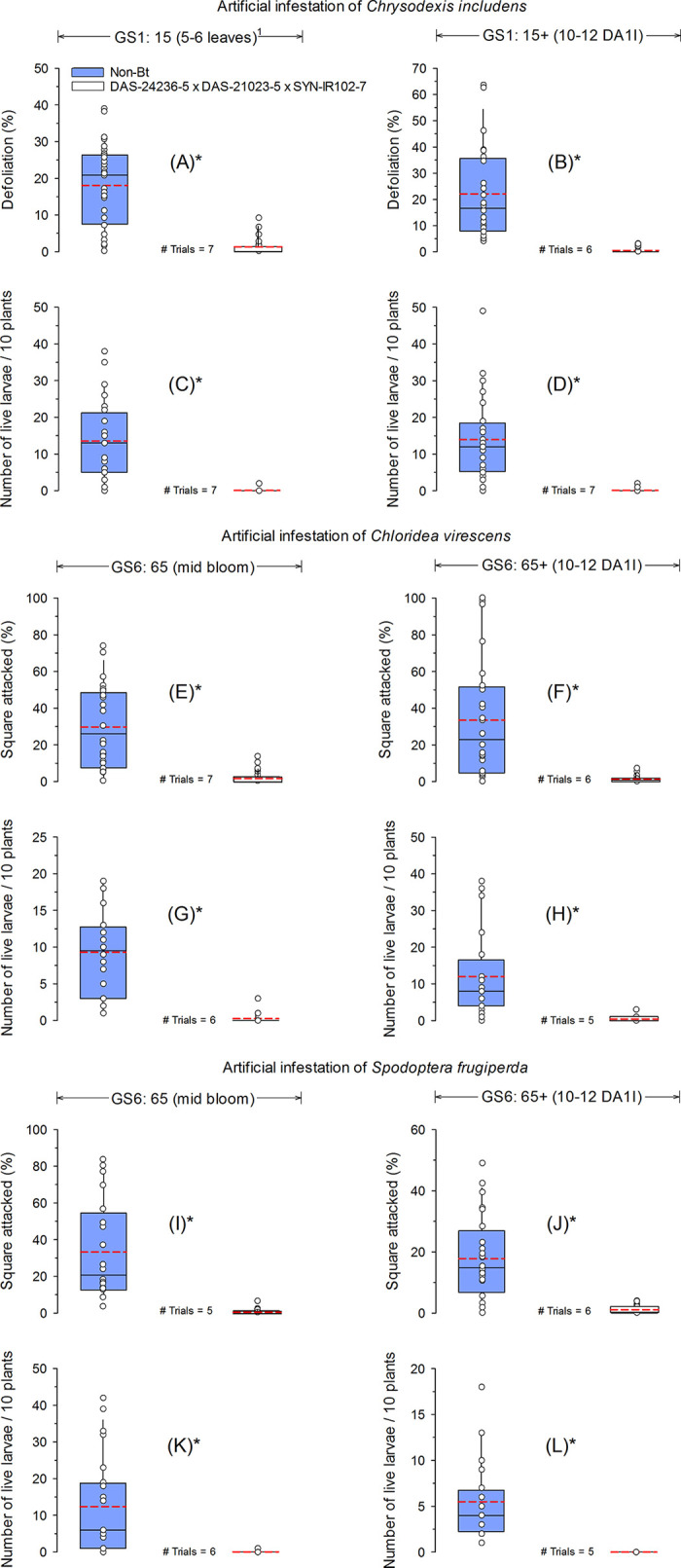
Efficacy of *Bt* cotton technology with events DAS-21023-5 × DAS-24236-5 × SYN-IR102-7 against *Chrysodeixis includens*, *Chloridea virescens* and *Spodoptera frugiperda* at 10 days after artificial infestation of first instar (L1) during vegetative and reproductive cotton growth stages. The dashed (red) and solid (black) lines in boxplots represent the mean and median across trials, respectively. Dot markers indicate values from individual trials. *Significant difference between non-*Bt* and *Bt* cotton technology using an *F*-test (α = 0.05). ^1^Phenological growth stages of cotton classified according to Munger et al. (1998); DA1I = days after 1^st^ infestation.

Mean defoliation caused by *C*. *includens* was 1.3% for *Bt* cotton with 5–6 leaves and 0.5% for plants infested 10–12 days after the first infestation, while in non-*Bt* cotton (control) defoliation reached 18.1 and 22.1% in the first and second infestation, respectively (Fig 1A and 1B). Ten days after each infestation, the mean number of *C*. *includens* live larvae found on non-*Bt* cotton was approximately 14 larvae/10 plants. For DAS-21023-5 × DAS-24236-5 × SYN-IR102-7 *Bt* cotton, 0.1 larvae/10 plants were observed (Fig 1C and 1D). At the GS6:65 cotton growth stages, the percentage of squares attacked by *C*. *virescens* was 29.7% in non-*Bt* cotton and 1.9% in *Bt* cotton, while at GS6:65+ the percentage of squares attacked was 33.5 and 1.4% in the non-*Bt* and *Bt* cotton treatments, respectively (Fig 1E and 1F). After the first and second infestation, mean *C*. *virescens* live larvae found 10 DAI in non-*Bt* cotton was 9.3 and 12.0 larvae/10 plants, whereas in DAS-21023-5 × DAS-24236-5 × SYN-IR102-7 *Bt* cotton, only 0.3 and 0.4 larvae/10 plants were recovered (Fig 1G and 1H). Results from artificial infestations of *S*. *frugiperda* at cotton reproductive stages showed similar patterns as those of *C*. *virescens*. The percentage of non-*Bt* cotton squares attacked by *S*. *frugiperda* at GS6:65 and GS6:65+ cotton growth stages were 33.2 and 17.8%, respectively. During the same stages of *Bt* cotton development, only 0.8 and 0.9% of squares were attacked (Fig 1I and 1J). The mean number of *S*. *frugiperda* live larvae 10 DAI at the GS6:65 cotton growth stage was 12.4 and 0.8 larvae/10 plants in non-*Bt* and DAS-21023-5 ×DAS-24236-5 × SYN-IR102-7 *Bt* cotton, respectively. At GS6:65+ cotton growth stages, a mean of 5.5 *S*. *frugiperda* larvae were found in non-*Bt* cotton, while no live larvae were found on DAS-21023-5 × DAS-24236-5 × SYN-IR102-7 plants (Fig 1K and 1L).

**Fig 2:**
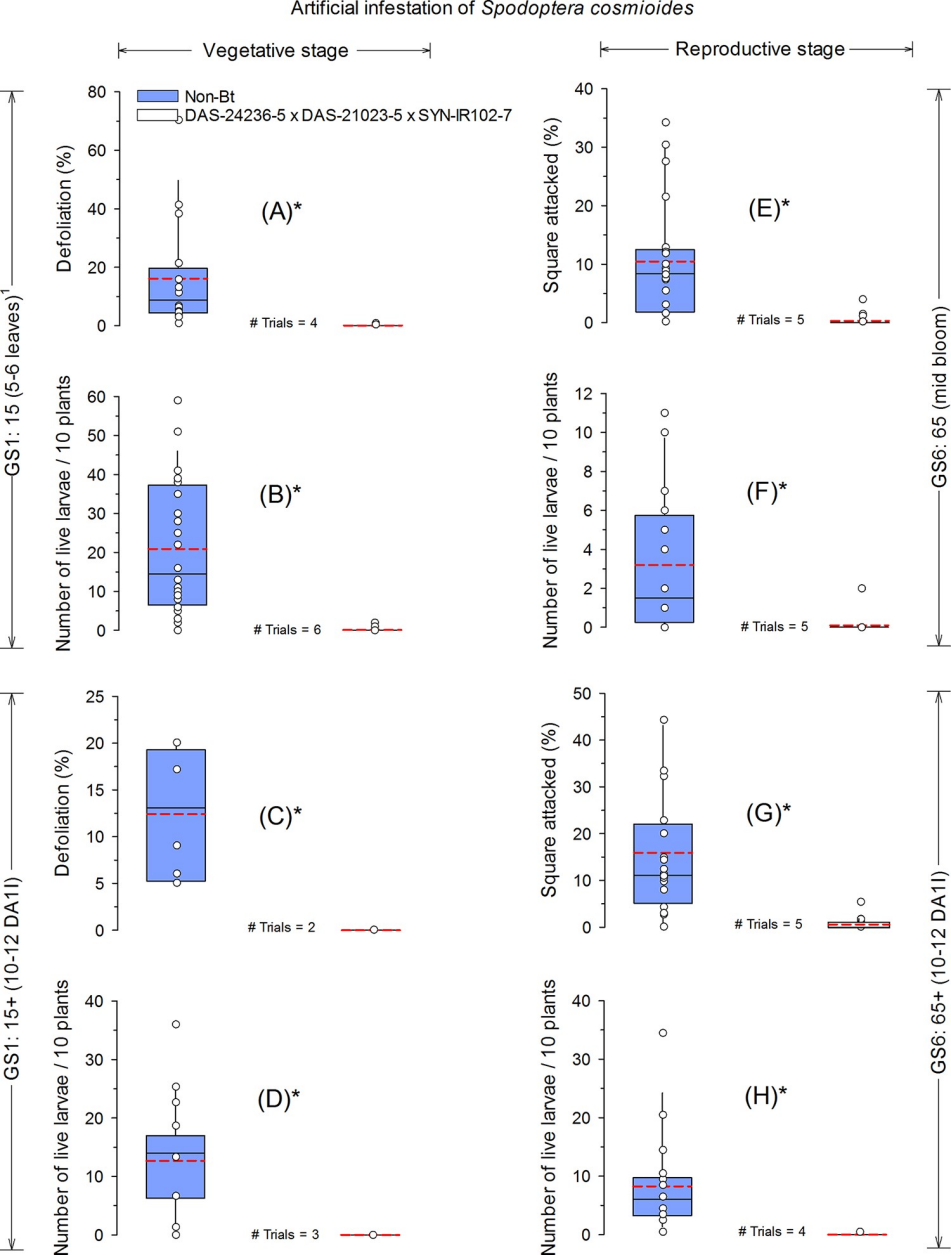
Efficacy of *Bt* cotton technology with events DAS-21023-5 × DAS-24236-5 × SYN-IR102-7 against *Spodoptera cosmioides* at 10 days after artificial infestations of first instar (L1) during vegetative and reproductive cotton growth stages. The dashed (red) and solid (black) lines in boxplots present the mean and median across trials, respectively. Dot markers indicate values from individual trials. *Significant difference between non-*Bt* and *Bt* cotton technology using an *F*-test (α = 0.05). ^1^Phenological growth stages of cotton classified according to Munger et al. (1998); DA1I = days after 1^st^ infestation.

DAS-21023-5 × DAS-24236-5 × SYN-IR102-7 *Bt* cotton demonstrated excellent efficacy against *S*. *cosmioides* in both vegetative and reproductive cotton (Fig 2). In the GS1:15 vegetative stage (5–6 leaves), the percentage of defoliation caused by *S*. *cosmioides* and the mean number of live larvae found in the non-*Bt* cotton were 16.1% and 20.9 larvae/10 plants, respectively. In the *Bt* cotton, these values were 0.1% and 0.1 larvae/10 plants, respectively (Fig 2A and 2B). After the second infestation of *S*. *cosmioides* in the vegetative stage (GS1:15+; 10–12 days after first infestation), the percentage of defoliation and mean live larvae in non-*Bt* cotton were 12.4% and 12.7 larvae/10 plants, while in *Bt* cotton, no defoliation was observed and no live larvae of *S*. *cosmioides* were found 10 days after infestation (Fig 2C and 2D). During the reproductive stage (GS6:65; mid bloom), the percentage of squares attacked by *S*. *cosmioides* and the number of live larvae found (10 DAI) in the non-*Bt* cotton were 10.5% and 3.2 larvae/10 plants, while in DAS-21023-5 × DAS-24236-5 × SYN-IR102-7 the observed means were 0.3% and 0.1 larvae/10 plants (Fig 2E and 2F). At GS6:65+, the percentage of squares injured in non-*Bt* plots was 15.9%, while in *Bt* cotton only 0.6% of squares were injured (Fig 2G). Ten days after infestation, the mean number of *S*. *cosmioides* larvae found in the non-*Bt* and *Bt* cotton were 8.3 and 0 larvae/10 plants, respectively (Fig 2H).

Based on the results from artificial infestations, DAS-21023-5 × DAS-24236-5 × SYN-IR102-7 *Bt* cotton significantly reduced injury caused by all lepidopteran species evaluated and caused high levels of mortality (cotton growth stages = percentage mean ± SE) on *S*. *frugiperda* (GS6:65 = 99.9 ± 0.0; GS6:65+ = 100), *S*. *cosmioides* (GS1:15 = 99.9 ± 0.4; GS1:15+ = 100; GS6:65 = 99.9 ± 0.1; GS6:65+ = 100), *C*. *includens* (GS1:15 = 99.9 ± 0.1; GS1:15+ = 99.8 ± 0.1) and *C*. *virescens* (GS6:65 = 99.8 ± 0.1; GS6:65+ = 99.6 ± 0.2).

#### Natural infestation

**Fig 3:**
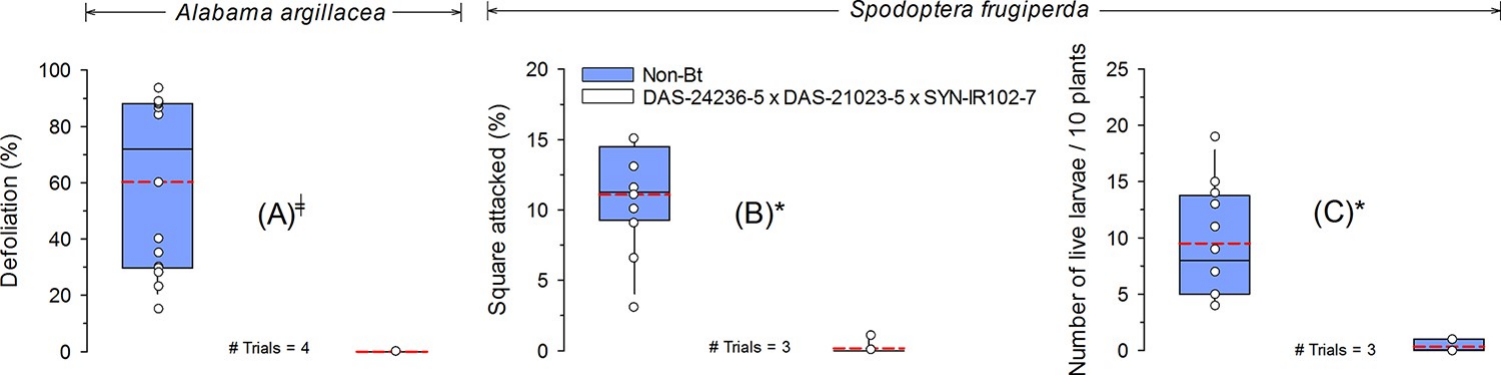
Efficacy of *Bt* cotton technology with events DAS-21023-5 × DAS-24236-5 × SYN-IR102-7 against natural infestations of *Alabama argillacea* or *Spodoptera frugiperda*. The dashed (red) and solid (black) lines in boxplots present the mean and median across trials, respectively. Dot markers indicate values from individual trials. ^╪^Statistical test not applied due to a numerical difference sufficiently large enough to declare a difference between treatments means. *Significant difference between non-*Bt* and *Bt* cotton technology by *F*-test (α = 0.05).

Under natural infestations of *A*. *argillacea*, *Bt* cotton demonstrated complete protection from defoliation (zero percent defoliation) compared with the non-*Bt* cotton which suffered 60.3% defoliation (Fig 3A). *Bt* cotton also significantly reduced the percentage of squares injured by *S*. *frugiperda* and the mean number of live larvae. The mean of squares injured and the mean number of *S*. *frugiperda* live larvae in the DAS-21023-5 × DAS-24236-5 × SYN-IR102-7 *Bt* cotton were 0.2% and 0.3 larvae/10 plants, respectively. In the non-*Bt* cotton, these values were 11.1% and 9.5 larvae/10 plants (Fig 3B and 3C).

### Effects on foliar-dwelling and non-target aerial arthropods

#### Beat cloth assessment

**Fig 4:**
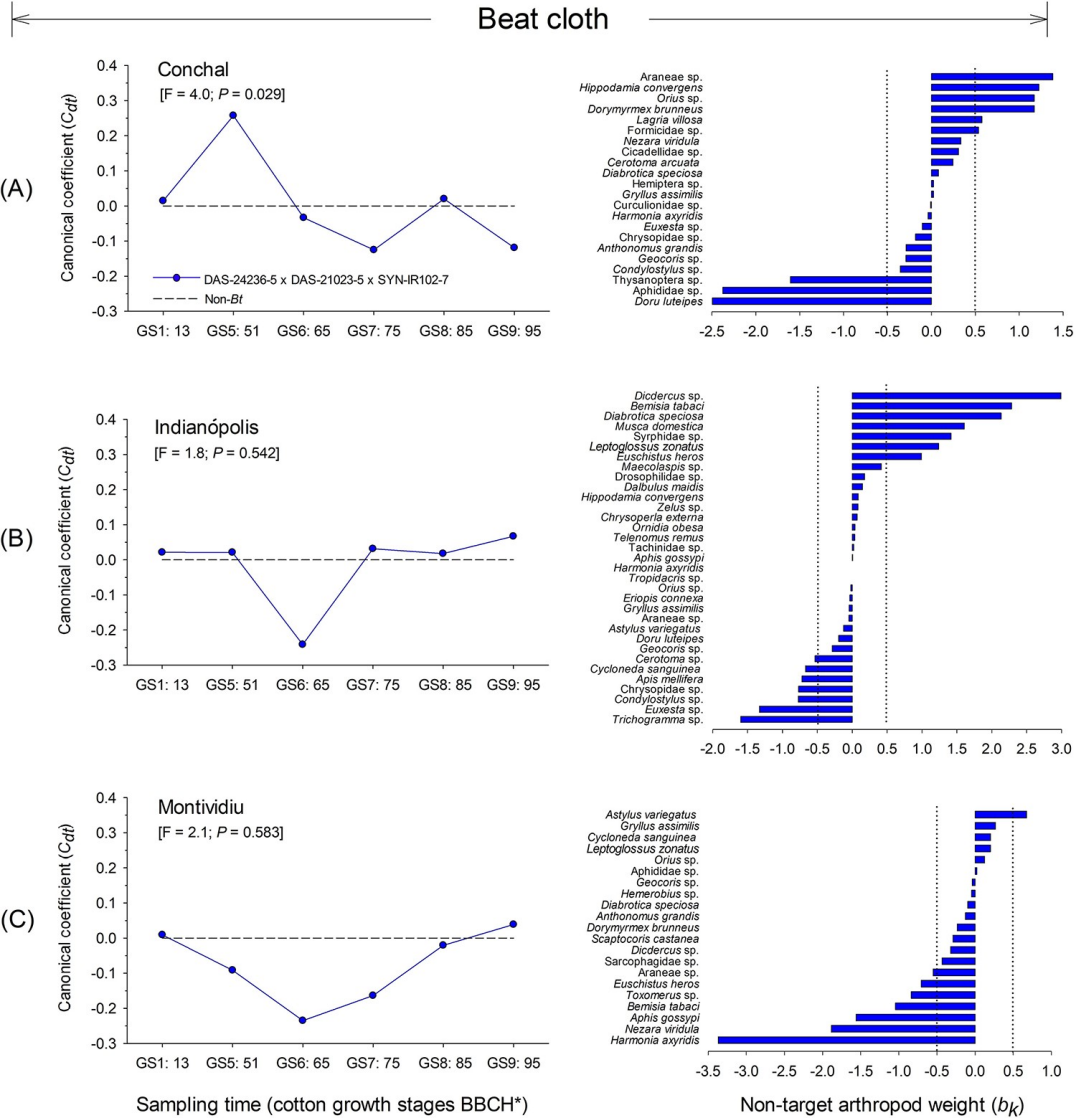
Principal response curves (PRCs) and taxon weights of foliar-dwelling arthropod populations collected via beat cloth sampling from DAS-21023-5 × DAS-24236-5 × SYN-IR102-7 *Bt* cotton compared with a non-*Bt* cotton variety at three sites in Brazil (2014/2015 cropping season). Dotted line indicates the response within the non-*Bt* cotton entry. If a significant *P-value* was detected (< 0.05, Monte Carlo test), taxa with positive weights followed the PRC pattern, whereas those with negative weights showed the opposite pattern. Taxa with weights between approximately -0.5 and 0.5 did not contribute significantly to the overall pattern. If a *P-value* was non-significant, species patterns were random in relation to treatments. *Phenological growth stages of cotton classified according to Munger et al. (1998).

The results of RDAs for the NTAs collected via the beat cloth method revealed significant differences between the DAS-21023-5 × DAS-24236-5 × SYN-IR102-7 *Bt* cotton technology and the non-*Bt* cotton (control) in one of the three trial locations (Conchal: F = 4.0; *P* = 0.029; Indianópolis: F = 1.8; *P* = 0.542 and Montividiu: F = 2.1; *P* = 0.583) (Fig 4). For the Conchal trial, the first axis of the RDA explained 41.2% of the total variation of the sampled community, within which 58.1% of the variance was associated with sampling time (cotton growth stage) and 2.1% associated with cotton type. In the PRC diagram for Conchal, DAS-21023-5 × DAS-24236-5 × SYN-IR102-7 presented positive canonical coefficient (*Cdt*) at the GS1:13, GS5:51 and GS8: 85 cotton growth stages (Fig 4A). Among the NTAs that contributed most to the community response in Conchal (taxon weights > |0.5|), the predators Araneae sp. (Arachnidae), *Hippodamia convergens* (Coleoptera: Coccinellidae), *Orius* sp. (Hemiptera: Anthocoridae), and omnivores *Dorymyrmex brunneus* (Hymenoptera: Formicidae) and Formicidae sp. (Hymenoptera) showed positive weights (*bk*) as well as the herbivorous beetle, *Lagria villosa* (Coleoptera: Lagriidae). These taxa followed the PRC trend by exhibiting higher abundance in DAS-21023-5 × DAS-24236-5 × SYN-IR102-7 plots at the GS5:51 cotton growth stage and lower abundance at the GS6:65, GS7:75 and GS9:95 cotton stages (Fig 4A). In contrast, the predator *Doru luteipes* (Dermaptera: Forficulidae) and herbivores from Aphididae sp. (Hemiptera) and Thysanoptera sp. exhibited negative taxon weights, and therefore lower abundance in DAS-21023-5 × DAS-24236-5 × SYN-IR102-7 plots at the GS5:51 cotton growth stage and an increased abundance at the GS6:65, GS7: 75 and GS9:95 cotton stages (Fig 4A). In the other two trial locations, no differences were detected.

**Fig 5:**
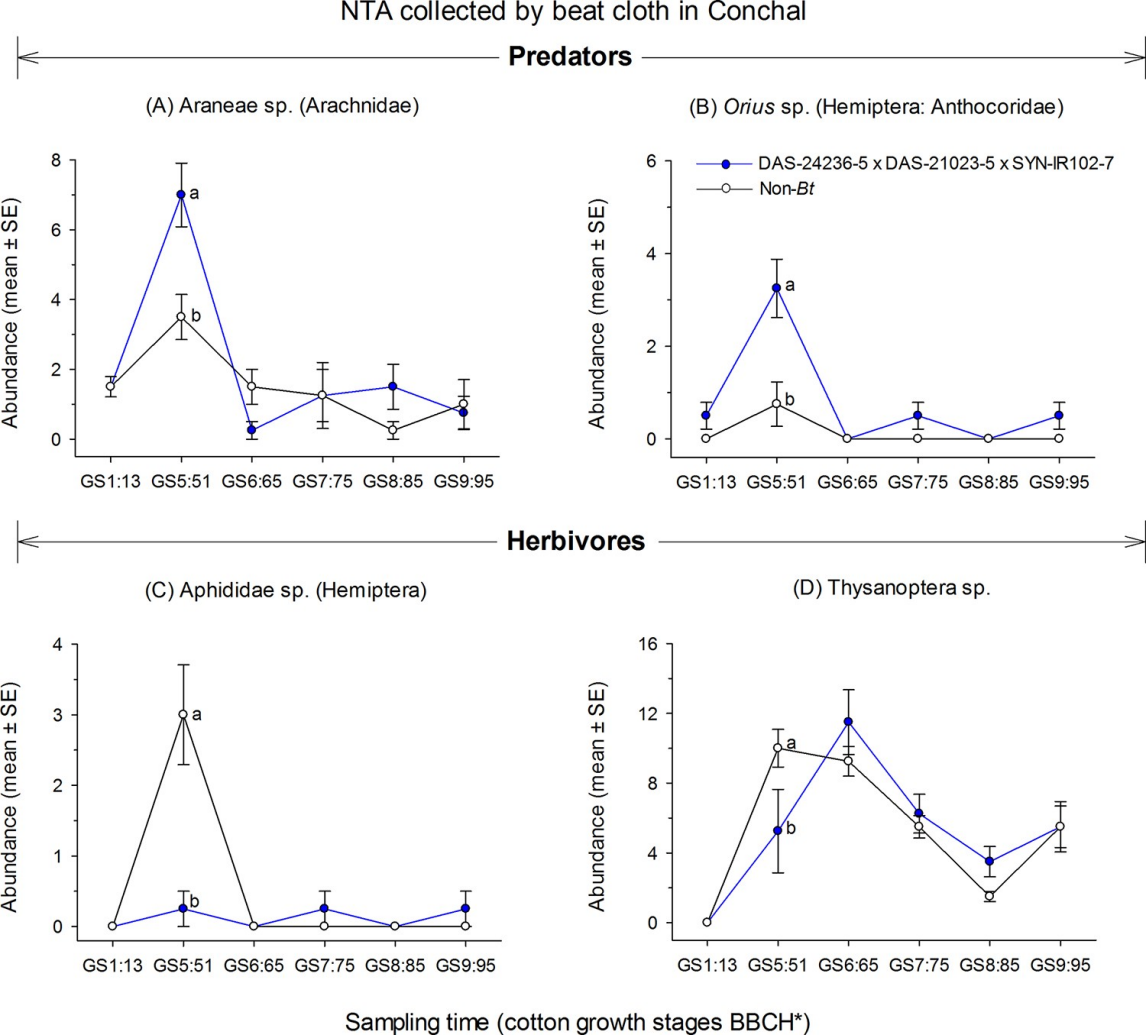
Abundance of foliar-dwelling arthropods collected via beat cloth method in a cotton trial at Conchal (2014/2015 cropping season). Means (± SE) within sampling time followed by different letters are significantly different by two-way RM-ANOVA test (α = 0.05). *Phenological growth stages of cotton classified according to Munger et al. (1998).

The results of two-way RM-ANOVA for taxa with PRC taxon weights > |0.5| indicated that the interaction effect of cotton type (DAS-21023-5 × DAS-24236-5 × SYN-IR102-7 *Bt* cotton and non-*Bt* cotton) and sampling time (cotton growth stage) was significant only for Araneae sp. (F = 4.1; df = 5, 15; *P* = 0.015), *Orius* sp. (F = 4.3; df = 5, 15; *P* = 0.013), Aphididae sp. (F = 16.4; df = 5, 15; *P* < 0.001) and Thysanoptera sp. (F = 4.9; df = 5, 15; *P* = 0.009) (Fig 5). For all other NTAs the interaction was not significant (S2 Table). The abundance of Araneae predators and *Orius* sp. during the GS5:51 cotton growth stage was higher in DAS-21023-5 × DAS-24236-5 × SYN-IR102-7 compared with the non-*Bt* cotton plots (Fig 5A and 5B). In contrast, the abundance of the herbivores from Aphididae and Thysanoptera during the GS5:51 sampling time were lower in DAS-21023-5 × DAS-24236-5 × SYN-IR102-7 plots (Fig 5C and 5D).

#### Sticky card trapping

**Fig 6:**
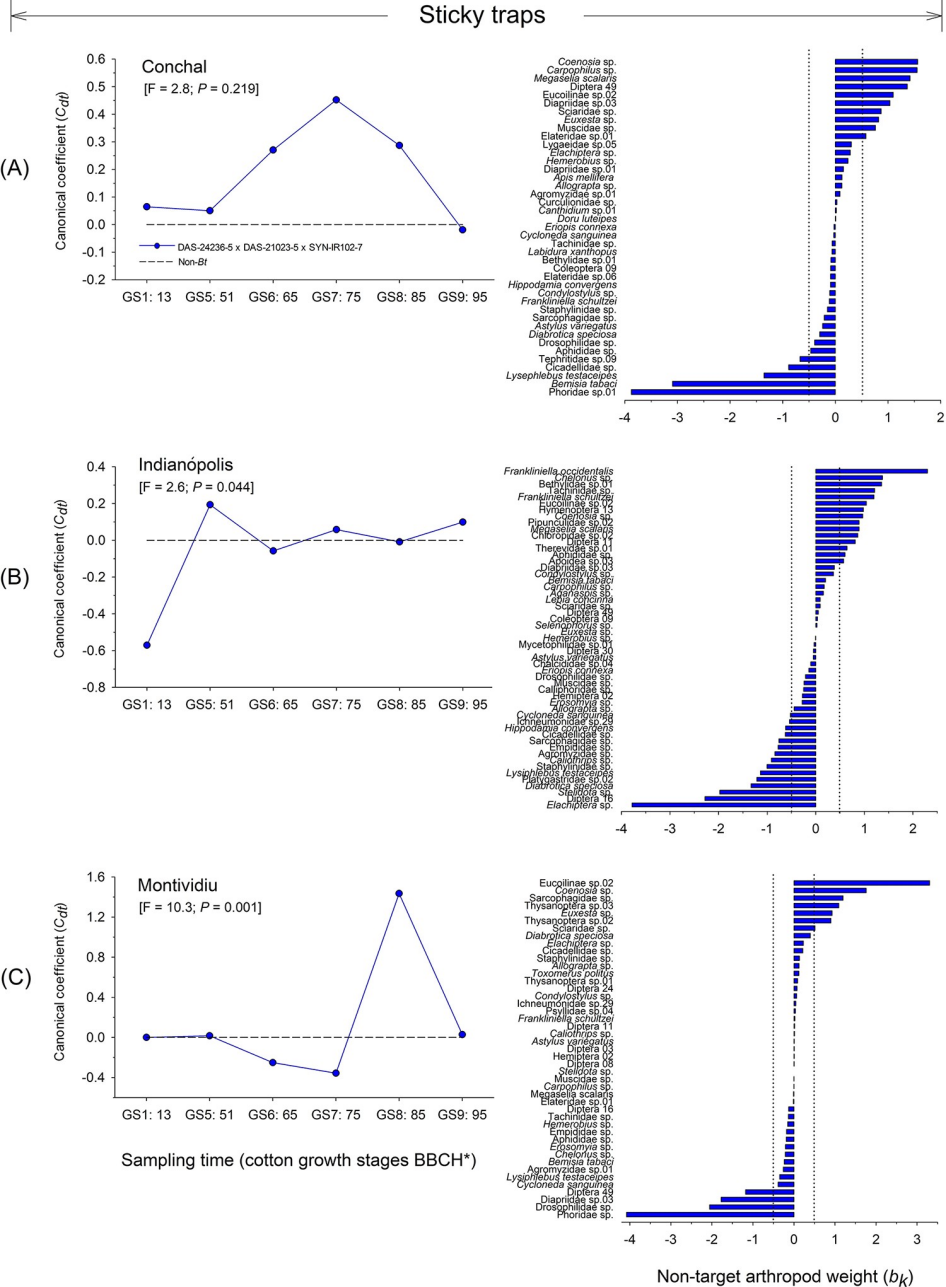
Principal response curves (PRCs) and taxon weights of aerial-dwelling arthropod populations collected via sticky card trapping from DAS-21023-5 × DAS-24236-5 × SYN-IR102-7 *Bt* cotton compared with a non-*Bt* variety at three sites in Brazil (2014/2015 cropping season). Dotted line indicates the response within the non-*Bt* cotton entry. If a significant *P-value* was detected (< 0.05, Monte Carlo test), taxa with positive weights followed the PRC pattern, whereas those with negative weights showed the opposite pattern. Taxa with weights between approximately -0.5 and 0.5 did not contribute significantly to the overall pattern. If a *P-value* was non-significant, species patterns were random in relation to treatments. *Phenological growth stages of cotton classified according to Munger et al. (1998).

For the NTA community sampled via sticky card traps at Conchal, the first axis was not significant (F = 2.8; *P* = 0.219) (Fig 6A). However, at Indianópolis (F = 2.6; *P* = 0.044) and Montividiu (F = 10.3; *P* = 0.001) the first axis was significant, indicating differences between populations associated with DAS-21023-5 × DAS-24236-5 × SYN-IR102-7 *Bt* cotton technology and non-*Bt* cotton at these sites (Fig 6B and 6C). At Indianópolis, the first axis explained 31.3% of the total variation of the sampled community, within which 79.2% of the variance was associated with sampling time and 0.8% associated with cotton type. The NTAs that most contributed to the community response at Indianópolis were the herbivore *Frankliniella occidentalis* (Thysanoptera: Thripidae) and the parasitoid fly *Elachiptera* sp. (Diptera: Chloropidae), with the highest and lowest taxon weights, respectively (Fig 6B).

At Montividiu, the first axis explained 30.1% of the total variation of NTAs community structure collected by sticky card traps, within which 73.1% of the variance was associated with sampling time and 0.9% associated with cotton type. The parasitoids Eucoilinae species 02 (Hymenoptera) and Phoridae sp. (Diptera) were the NTAs that most contributed to the community response, with the highest and lowest weights, respectively (Fig 6C).

**Fig 7:**
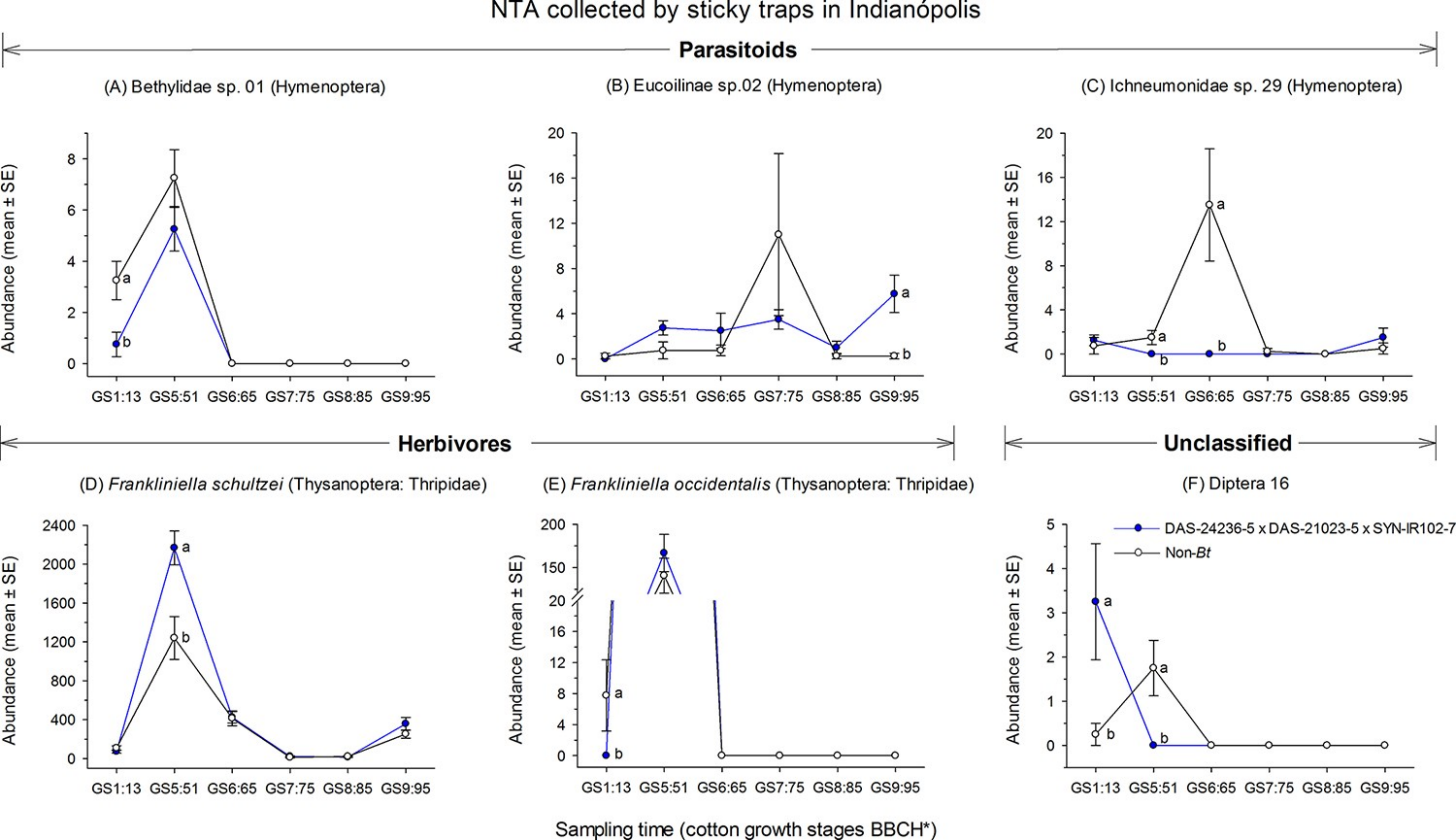
Abundance of aerial-dwelling arthropods collected via sticky card trapping in a cotton trial at Indianópolis (2014/2015 cropping season). Means (± SE) within sampling time followed by different letters are significantly different by two-way RM-ANOVA (α = 0.05). *Phenological growth stages of cotton classified according to Munger et al. (1998).

Where a significant Monte Carlo test indicated potential differences for NTAs sampled using sticky card traps at Indianópolis, a two-way RM-ANOVA for taxa with weights (*bk*) > |0.5| identified that Bethylidae species 01 (Hymenoptera) (F = 7.1; df = 5, 15; *P* = 0.001), Eucoilinae species 02 (F = 4.0; df = 5, 15; *P* = 0.016), Ichneumonidae species 29 (Hymenoptera) (F = 8.6; df = 5, 15; *P* < 0.001), *Frankliniella schultzei* (Thysanoptera: Thripidae) (F = 3.2; df = 5, 15; *P* = 0.038), *F*. *occidentalis* (F = 3.2; df = 5, 15; *P* = 0.037) and Diptera morphotype 16 (F = 7.8; df = 5, 15; *P* < 0.001) showed significant interaction between cotton type and sampling time (S2 Table, Fig 7). The abundance of the hymenopteran parasitoids Bethylidae sp. 01 at the GS5:51 cotton growth stage and Ichneumonidae sp. 29 during the GS5:51 and GS6:65 cotton stages were lower in DAS-21023-5 × DAS-24236-5 × SYN-IR102-7 plots compared with the non-*Bt* cotton plots. In contrast, the abundance of Eucoilinae sp. 02 was significantly higher in DAS-21023-5 × DAS-24236-5 × SYN-IR102-7 plots at the GS9:95 sampling time (Fig 7A–7C). The abundance of *F*. *schultzei* at the GS5:51 cotton growth stage was significantly higher in DAS-21023-5 × DAS-24236-5 × SYN-IR102-7 plots compared with the non-*Bt* cotton plots. However, for *F*. *occidentalis* the abundance in DAS-21023-5 × DAS-24236-5 × SYN-IR102-7 plots at the GS1:13 sampling time was significantly lower in non-*Bt* cotton plots (Fig 7D and 7E). The abundance of Diptera sp. 16 was significantly higher in DAS-21023-5 × DAS-24236-5 × SYN-IR102-7 plots than non-*Bt* cotton plot during GS1:13 and then the opposite trend was observed during GS5:51 (Fig 7F).

**Fig 8:**
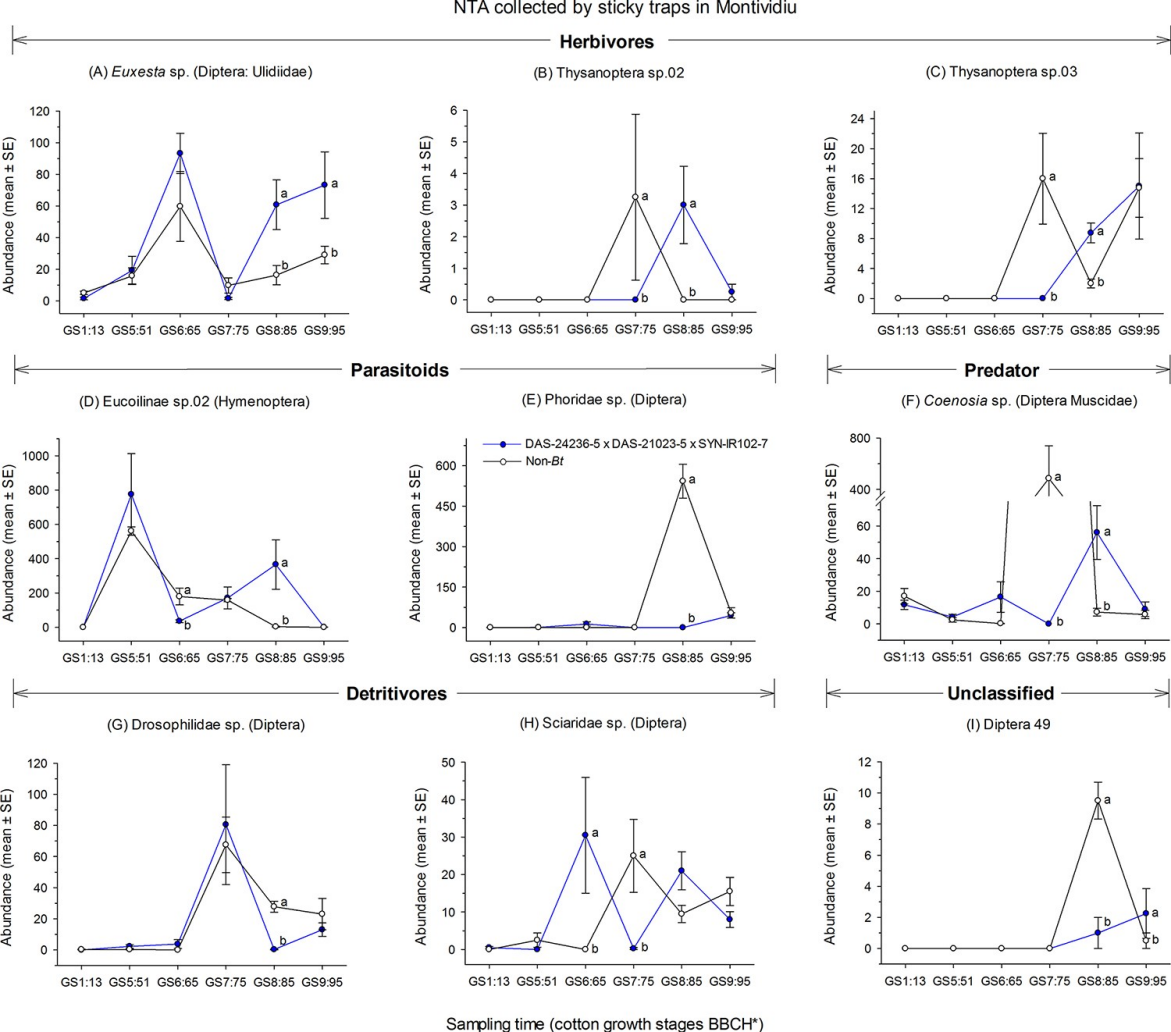
Abundance of aerial-dwelling arthropods collected via sticky card trapping in a cotton trial at Montividiu (2014/2015 cropping season). Means (± SE) within sampling time followed by different letters are significantly different by two-way RM-ANOVA test (α = 0.05). *Phenological growth stages of cotton classified according to Munger et al. (1998).

At Montividiu, two-way RM-ANOVAs for NTAs with weights >|0.5| indicated statistical significance for the thrips, Thysanoptera species 02 (F = 5.4; df = 5, 15; *P* = 0.005) and Thysanoptera species 03 (F = 5.8; df = 5, 15; *P* = 0.003), the hymenopteran Eucoilinae sp. 02 (F = 44.9; df = 5, 15; *P* < 0.001) and the dipterans *Euxesta* sp. (F = 4.7 df = 5, 15; *P* = 0.009), Phoridae sp. (F = 65.8; df = 5, 15; *P* < 0.001), *Coenosia* sp. (F = 12.7; df = 5, 15; *P* < 0.001), Drosophilidae sp. (F = 16.8; df = 5, 15; *P* < 0.001), Sciaridae sp. (F = 10.8; df = 5, 15; *P* < 0.001) and Diptera morphotype 49 (F = 12.4; df = 5, 15; *P* < 0.001) (S2 Table, Fig 8). In general, except for Sciaridae, differences in NTAs abundance in DAS-21023-5 × DAS-24236-5 × SYN-IR102-7 plots and non-*Bt* cotton were at the GS8:85 cotton growth stage. The abundance of *Euxesta* sp. during the GS8:85 and GS9:95 cotton growth stages were significant higher in DAS-21023-5 × DAS-24236-5 × SYN-IR102-7 plots compared with non-*Bt* cotton (Fig 8A). The thrips, Thysanoptera sp. 02 and 03 were more abundant in non-*Bt* cotton and DAS-21023-5 × DAS-24236-5 × SYN-IR102-7 plots during the GS7:75 and GS8:85, respectively (Fig 8B and 8C). The abundance of Eucoilinae sp. 02 in DAS-21023-5 × DAS-24236-5 × SYN-IR102-7 plots compared with non-*Bt* cotton was significant lower in GS6:65 and higher during GS8:85, while phorid flies in non-*Bt* cotton plots were more abundant during GS8:85 (Fig 8D and 8E). The predator *Coenosia* sp. was more abundant in non-*Bt* cotton at GS7:75 compared with DAS-21023-5 × DAS-24236-5 × SYN-IR102-7 plots. However, during the GS8:85 cotton growth stage, *Coenosia* sp. abundance was significantly lower in non-*Bt* cotton plots (Fig 8F). In DAS-21023-5 × DAS-24236-5 × SYN-IR102-7 plots, the detritivore-containing fly families, Drosophilidae and Sciaridae, were less abundant compared with non-*Bt* cotton at the GS8:85 and GS7:75 sampling times, respectively. However, Sciaridae sp. at the GS6:65 cotton growth stage was more abundant in DAS-21023-5 × DAS-24236-5 × SYN-IR102-7 plots (Fig 8G and 8H). For the Diptera morphotype 49, abundance was higher during the GS8:85 cotton growth stage in non-*Bt* cotton plots. However, the reverse trend was observed in the subsequent sampling period (GS9:95) (Fig 8I).

### Effects on ground-dwelling non-target arthropods

#### Pitfall trapping

**Fig 9:**
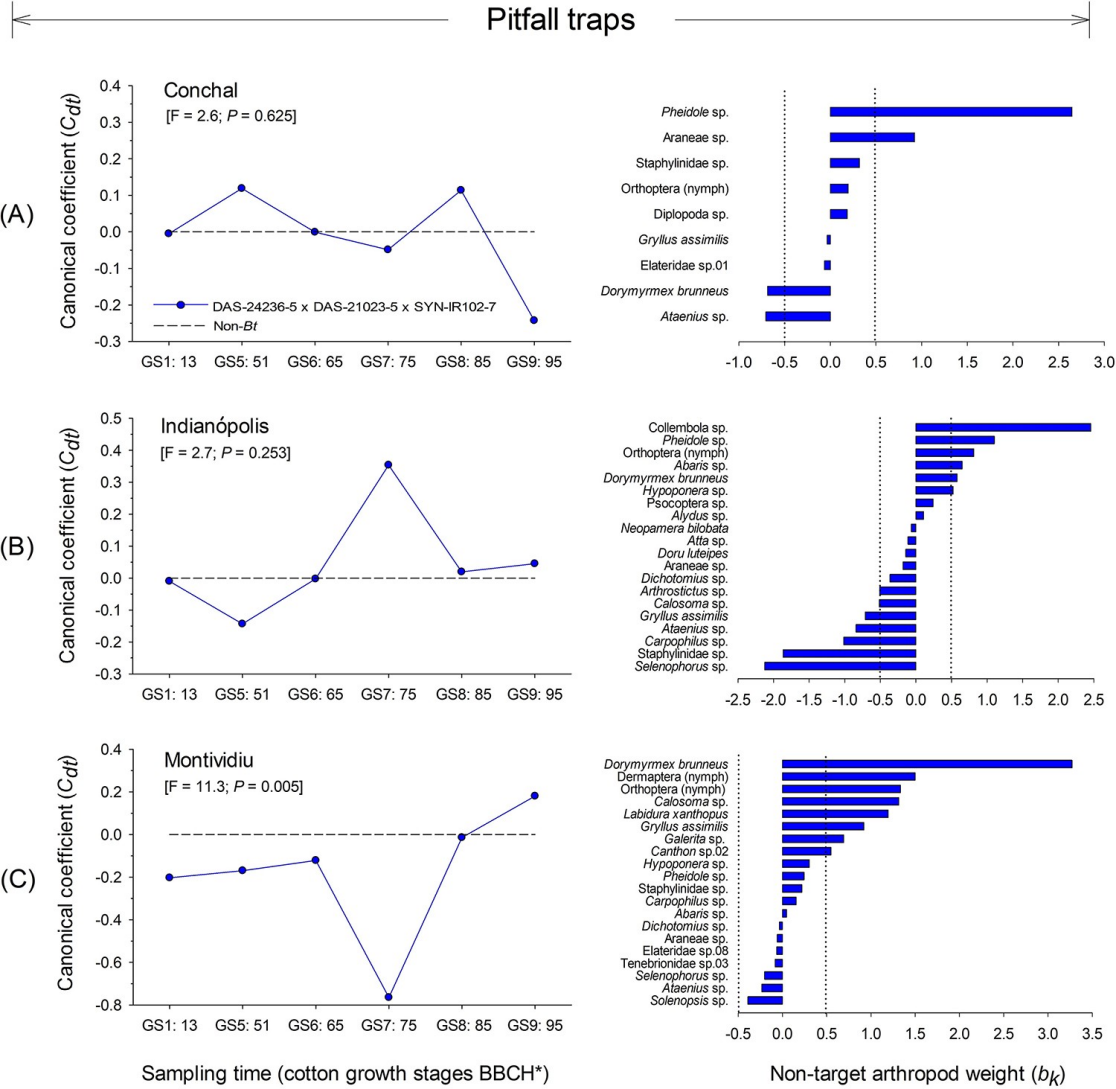
Principal response curves (PRCs) and taxon weights of ground-dwelling arthropod populations collected via pitfall trapping from DAS-21023-5 × DAS-24236-5 × SYN-IR102-7 *Bt* cotton compared with a non-*Bt* variety at three sites in Brazil (2014/2015 cropping season). Dotted line indicates the response within the non-*Bt* cotton entry. If a significant *P-value* was detected (< 0.05, Monte Carlo test), taxa with positive weights followed the PRC pattern, whereas those with negative weights showed the opposite pattern. Taxa with weights between approximately -0.5 and 0.5 did not contribute significantly to the overall pattern. If a *P-value* was non-significant, species patterns were random in relation to treatments. *Phenological growth stages of cotton classified according to Munger et al. (1998).

For the NTA community sampled via pitfall trapping, the first axis in RDA was not significant at Conchal (F = 2.6; *P* = 0.625) or Indianópolis (F = 2.7; *P* = 0.253) (Fig 9A and 9B). At Montividiu, the first axis of RDA was significant (F = 11.3; *P* = 0.005) and explained 75.7% of the total variation of NTAs community structure collected by pitfall traps, within which 48.7% of the variance was associated with sampling time and 4.0% associated with cotton type (Fig 9C).

**Fig 10:**
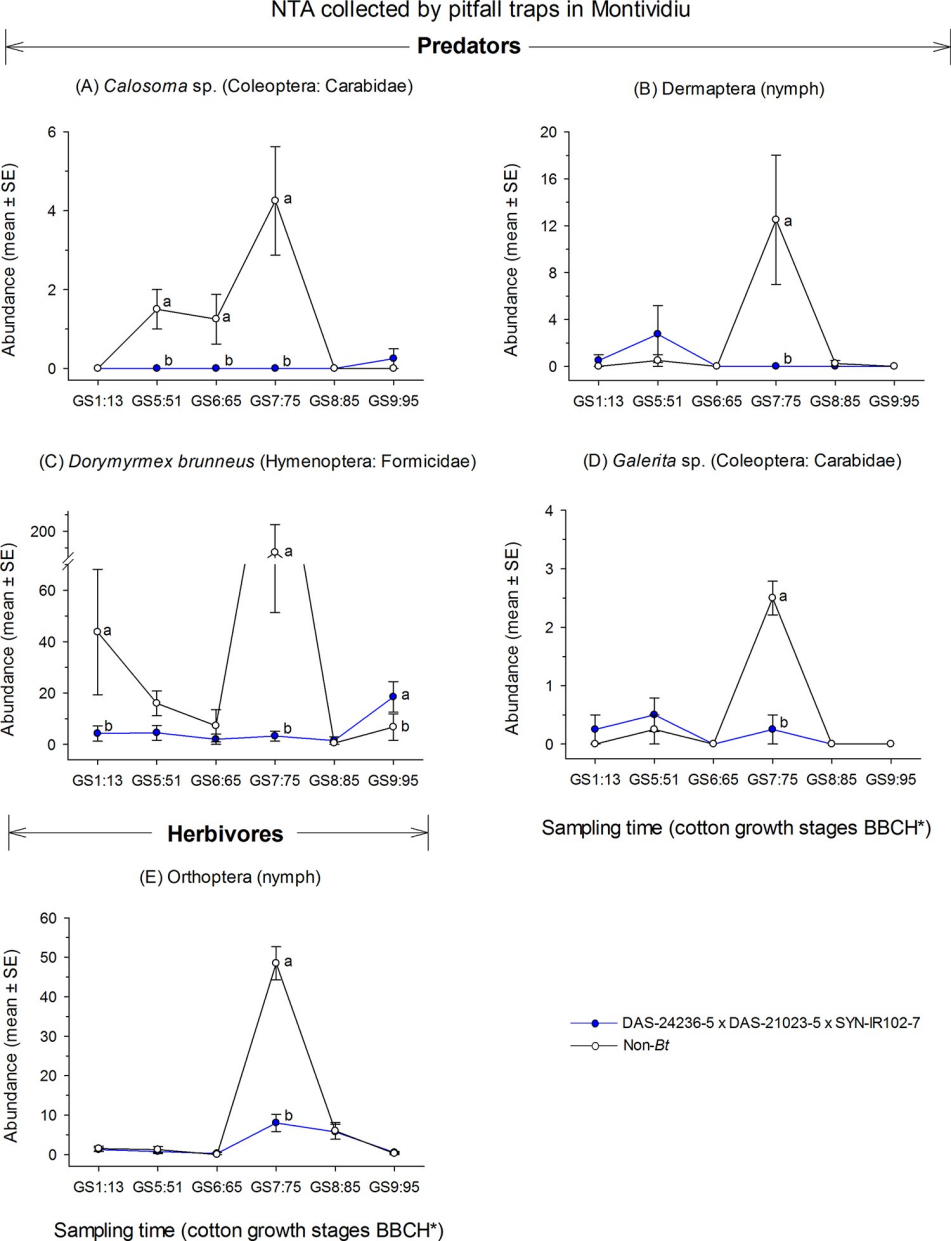
Abundance of ground-dwelling arthropods collected via pitfall trapping in a cotton trial at Montividiu (2014/2015 cropping season). Means (± SE) within sampling time followed by different letters are significantly different by two-way RM-ANOVA (α = 0.05). *Phenological growth stages of cotton classified according to Munger et al. (1998).

The two-way RM-ANOVAs analyses for NTAs with weights >|0.5| identified statistically significant differences for *Calosoma* sp. (F = 9.8; df = 5, 15; *P* < 0.001), Dermaptera (nymph) (F = 7.8; df = 5, 15; *P* < 0.001), *D*. *brunneus* (F = 8.2; df = 5, 15; *P* < 0.001), *Galerita* sp. (Coleoptera: Carabidae) (F = 26.3; df = 5, 15; *P* < 0.001) and Orthoptera (nymph) (F = 6.8; df = 5, 15; *P* = 0.002) (S2 Table, Fig 10), at Montividiu. The abundance of these NTAs at the GS7:75 cotton growth stage was lower for DAS-21023-5 × DAS-24236-5 × SYN-IR102-7 plots compared with non-*Bt* cotton (Fig 10). The abundance of the predatory beetle *Calosoma* sp. at the GS5:51 and GS6:65 cotton growth stages and the ant *D*. *brunneus* at the GS1:13 cotton stage were also significantly lower for DAS-21023-5 × DAS-24236-5 × SYN-IR102-7 plots (Fig 10A and 10C). However, for the last sampling time the abundance of *D*. *brunneus* was higher in DAS-21023-5 × DAS-24236-5 × SYN-IR102-7 plots compared with non-*Bt* cotton (Fig 10C).

#### Berlese-Tullgren funnel soil extraction

**Fig 11:**
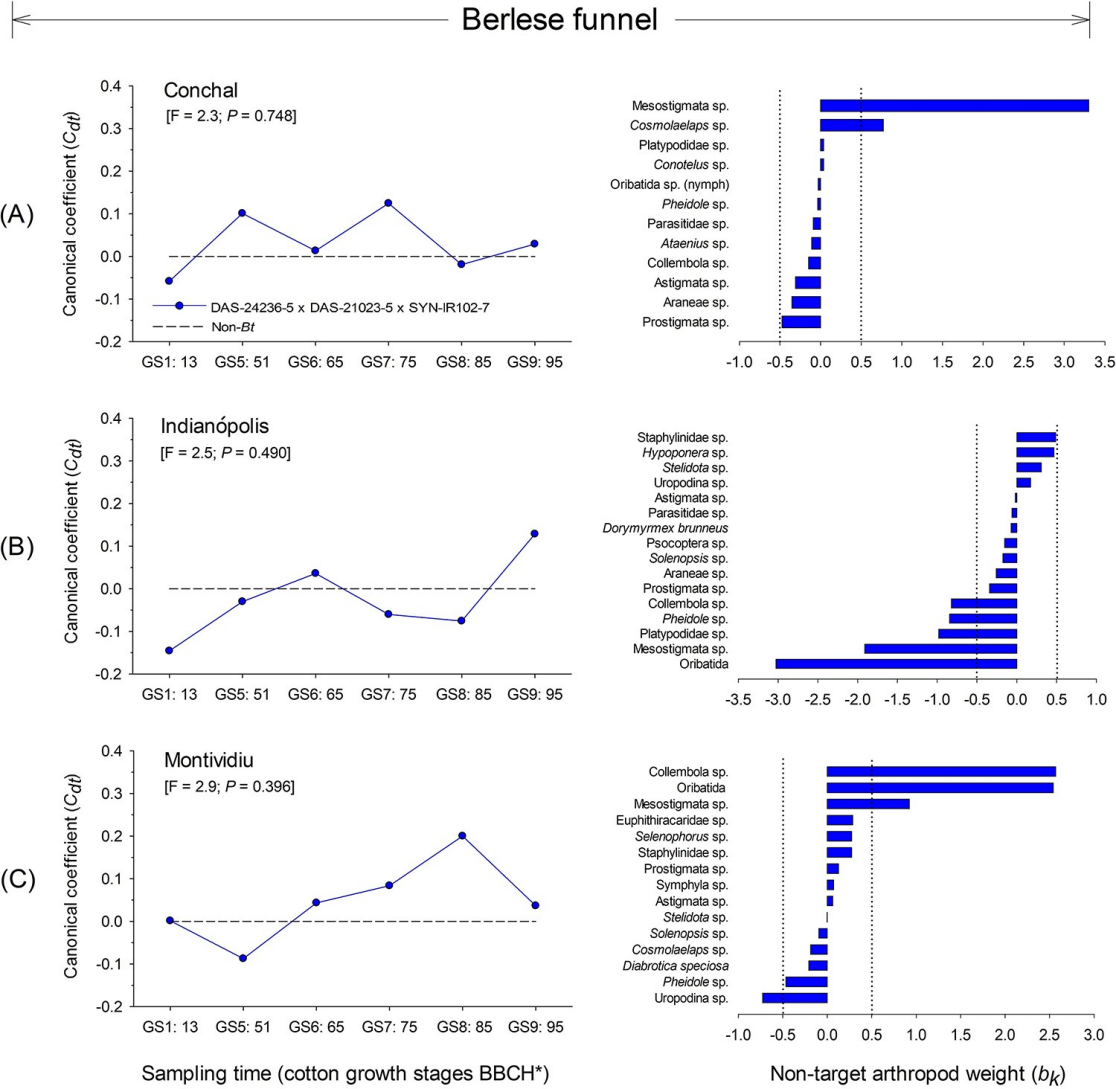
Principal response curves (PRCs) and taxon weights of ground-dwelling arthropod populations collected via soil extraction from DAS-21023-5 × DAS-24236-5 × SYN-IR102-7 *Bt* cotton compared with a non-*Bt* variety at three sites in Brazil (2014/2015 cropping season). Dotted line indicates the response within the non-*Bt* cotton entry. If a significant *P-value* was detected (< 0.05, Monte Carlo test), taxa with positive weights followed the PRC pattern, whereas those with negative weights showed the opposite pattern. Taxa with weights between approximately -0.5 and 0.5 did not contribute significantly to the overall pattern. If a *P-value* was non-significant, species patterns were random in relation to treatments. *Phenological growth stages of cotton classified according to Munger et al. (1998).

The RDA for NTAs collected using the Berlese-Tullgren funnel soil extraction method did not identify significant differences at any of the sites in the study: Conchal (F = 2.3; *P* = 0.748), Indianópolis (F = 2.5; *P* = 0.490) and Montividiu (F = 2.9; *P* = 0.396) (Fig 11). High taxon weights for the mite groups, Mesostigmata sp. and *Cosmolaelaps* sp. at Conchal (Fig 11A), and Oribatida and Mesostigmata sp., along with the beetle from Platypodidae sp., ant *Pheidole* sp. and Collembola sp. at Indianópolis (Fig 11B), and Collembola, Oribatida and Mesostigmata sp. at Montividiu (Fig 11C) identified common groups dominant in the soil environment.

## Discussion

The efficacy of the combined Cry1F, Cry1Ac and Vip3Aa19 insecticidal proteins expressed by DAS-21023-5 × DAS-24236-5 × SYN-IR102-7 *Bt* cotton in this study was characterized under a range of field conditions in several Brazilian cotton-growing regions. *Bt* cotton plants exhibited protection against *S*. *frugiperda*, *S*. *cosmioides*, *C*. *includens*, *C*. *virescens* and *A*. *argillacea* during vegetative and reproductive stages of cotton development. DAS-21023-5 × DAS-24236-5 × SYN-IR102-7 *Bt* cotton significantly reduced injury caused by S. *frugiperda*, *S*. *cosmioides*, *C*. *includens*, *C*. *virescens* and *A*. *argillacea* and caused high levels of mortality to all lepidopteran species evaluated, suggesting that survival to adult in the field might be reduced.

*Alabama argillacea*, *C*. *virescens*, *S*. *frugiperda* and *C*. *includens* are pests of cotton that can cause considerable injury [46–54]. In South America, outbreaks of *S*. *cosmioides*, which cause defoliation, have frequently been reported in cotton crops in Brazil [4, 55–57]. Our studies demonstrate that DAS-21023-5 × DAS-24236-5 × SYN-IR102-7 *Bt* cotton provided consistent protection from injury by the lepidopteran pest complex studied.

Our results using transgenic cotton containing three *Bt* proteins (Cry1F, Cry1Ac and Vip3Aa19) also support the work from previous authors evaluating dual *Bt* protein technologies in cotton. Siebert et al. [49] indicated Phytogen 440W containing Cry1Ac and Cry1F provided consistent control of heliothines across a range of environments and infestation levels in the southern United States. Another study in the same region showed that *C*. *virescens* was more susceptible to a transgenic cotton line expressing both Cry1Ab and Vip3A *Bt* proteins compared with a cotton line expressing only the Vip3A protein. Survivorship of *C*. *virescens* larvae was measured after feeding exposure to vegetative (terminal leaves) and reproductive (flower) structures of transgenic cotton. Survivorship ranged from 10 to 43% in Vip3A and from 2 to 12% in the dual *Bt* protein cotton expressing Cry1Ab and Vip3A, demonstrating the increased efficacy of the dual protein expression [58, 59]. Tindall et al. [50] reported that cotton plants containing Cry1Ac and Cry1F conferred high levels (100%) of soybean looper mortality and low levels (0.2%) of leaf defoliation compared with non-*Bt* cotton. Sorgatto et al. [60] reported high efficacy of cotton expressing Cry1Ac and Cry1F proteins against neonates of *C*. *includens*. Larvae of *S*. *cosmioides* reared on Cry1Ac and Cry1F cotton leaves exhibited reduced larval weight and did not reach the pupal stage [61].

In recent years, Brazilian cotton and maize fields have experienced more frequent high abundance *S*. *frugiperda* infestations, leading to significant economic losses [62, 63]. *Bt* cotton expressing Cry1Ac and Cry1F proteins required supplemental control with 5–8 foliar insecticide applications in Mato Grosso state [63]. Laboratory leaf disc bioassays using Cry1Ac and Cry1F cotton leaf tissue revealed a survival rate of 85% for *S*. *frugiperda* [63]. The high survival of *S*. *frugiperda* on Cry1Ac and Cry1F cotton is attributed to the low toxicity of Cry1Ac against this pest [64–67]. Additionally, *S*. *frugiperda* field-evolved resistance to Cry1F has been reported [68, 69]. The level of protection of Cry1F + Cry1Ac + Vip3Aa19 cotton (expressed with events DAS-21023-5 × DAS-24236-5 × SYN-IR102-7) in our studies against *S*. *frugiperda* was likely due to the high activity of Vip3Aa19 on this pest and agrees with previous studies testing a *Bt* cotton line expressing a single Vip3A protein [70]. Therefore, the new *Bt* cotton technology expressing the Cry1Ac + Cry1F + Vip3Aa19 *Bt* proteins will be an important tool that expands the range of control on a key lepidopteran pest complex. This technology should be deployed within the context of an IPM program and used with other locally defined best management practices and refuge requirements.

Our results of the present multi-site field study conducted under neotropical conditions also demonstrated that non-target arthropods (NTAs) associated with cotton in Brazil were not adversely affected by the *Bt* cotton technology expressing transgenic events DAS-21023-5 × DAS-24236-5 × SYN-IR102-7. The Principal Response Curve (PRC) method used to examine arthropod data for differences between cotton types indicated few and often minor differences in arthropod composition or abundance. At sites where the PRC method detected significant effects, the major differences were attributed to cotton growth stage (sampling time). These results were expected, as crop phenology is a key factor influencing arthropod community composition via the availability of resources [71]. Similar results were reported by Marques et al. [72], who observed that differences in the NTA community among fields of non-*Bt* soybean (with and without foliar insecticide applications), and *Bt* soybean expressing Cry1Ac and Cry1F proteins, were mostly related to sampling date.

In the present study, the NTA community associated with *Bt* cotton expressing transgenic events DAS-21023-5 × DAS-24236-5 × SYN-IR102-7 was indiscernible from that of non-*Bt* cotton. The similarity is further evidenced by the taxa that contributed most to the community response (weights (*bk*) > |0.5|) and even on those that showed a significant response in RM-ANOVA analysis (S2 Table), as differences were within the range of biological variation expected for those abundant and highly variable groups. Although many arthropod pests are associated with the cotton crop, their agroecosystem also has a high diversity of natural enemies [73] that are important biocontrol agents for maintaining pest populations at or below economic threshold levels [3, 74]. At Conchal, when the cotton first floral buds were detectable (GS5: 51 stage), the abundance of the predators from the order Araneae and *Orius* sp. (flower bug) collected by beat cloth were higher in *Bt* cotton, while the abundance of the herbivores from Aphididae and Thysanoptera were lower, compared with the non-*Bt* cotton. These results highlight the biological variation within arthropod populations and offer a snapshot of dynamics driven by factors other than cotton type. These results are consistent with the action of spiders and flower bugs as biological control agents for aphids and thrips in cotton. Aphids and thrips are not affected by the *Bt* cotton proteins tested in this study [13, 75]. Thus, complementary pest management tactics such as biological control is compatible with the use of this *Bt* cotton technology and well-timed crop protection insecticide applications based on locally-developed thresholds to support sustainable IPM.

Several predators are associated with arthropod pests of cotton, and the most common related to pest control in cotton include ants, stink bugs, lady beetles, lacewings and several species of spiders [3]. There is little information on the impact of natural enemies on thrips populations occurring on cotton seedlings [76]. However, several species of *Orius* have been shown to be effective predators of thrips species [77–79], although generally with low efficacy in suppressing thrips populations in cotton flowers [80]. Tian et al. [81] reported that *Bt* crops benefit from complementing action by natural enemies to control non-target pests such as aphids. These authors concluded that *Bt* plants expressing Cry1Ac and Cry1C do not impact predators and parasitoids of aphids, thus demonstrating the safety of these *Bt* plants in an IPM program. In two of the three study sites from our study, no differences in ground-dwelling arthropod fauna were observed between treatments. At Montividiu, when about 50% of cotton bolls attained their final size (GS7: 75 stage), ground-dwelling carabids, ants and orthopteran nymphs collected by pitfall trapping were more abundant in non-*Bt* cotton, compared with *Bt* cotton expressing *Bt* events DAS-21023-5 × DAS-24236-5 × SYN-IR102-7. Contrary to these results, *Bt* maize expressing different toxins Cry1Ab, Cry1F, Cry1A.105 and Cry2Ab2 did not affect the composition of ants and ground beetles [82]. At Indianópolis and Montividiu, sticky card monitoring showed similarities in NTA abundance and composition during the sampled periods, with observation of statistically significant differences in abundance only for some groups. The NTAs that contributed most to the community response at these sites (thrips and parasitoid wasps), showed temporarily higher abundance in non-*Bt* plots than in *Bt* plots during one of the six cotton stages surveyed. Where these isolated statistical differences were detected, patterns were observed at only one of the study sites, indicating observations were due to non-treatment factors and not expression of traits providing protection from insect pests or tolerance to herbicides. Furthermore, random differences in population abundance are common in NTA field studies as heterogeneously distributed populations are sampled as a point-estimate in time during their active periods. An additional consideration for *Bt* crop systems in particular is that the removal of the primary herbivorous pest (or host) can result in the absence of generalist natural enemies (or specialist parasitoids) that broadly suppress crop pests. A subsequent effect is the emergence of secondary, non-target pests whose populations may then fluctuate in size under lower and less consistent predation pressure. This may create the additional opportunity to detect higher variation in abundance, and random differences, of those secondary pests which may have been the case for observations at Indianópolis and Montividiu where common thrips species are typically numerically dominant (i.e., *F*. *schultzei* and *F*. *occidentalis*) but economically sub-dominant to the lepidopteran complex. For NTAs collected using Berlese-Tullgren funnel soil extraction, no difference was observed between the non-*Bt* and *Bt* cotton expressing the events DAS-21023-5 × DAS-24236-5 × SYN-IR102-7 at the three sites.

This study contributes broadly to the literature examining the potential impact of pest management strategies (here, *Bt* crops) on NTAs for the assessment of environmental risk [83] and increases the data for Brazil at the community level. Previous research carried out on a soybean crop in the USA [84] and in Brazil [72], on cotton in Australia [85] and maize in Europe [86, 87] and in China [88, 89] suggest that the responsible use of *Bt* crops has little or no influence on the NTA community. In a meta-analysis, Wolfenbarger et al. [35] also concluded that the application of insecticides in cotton, maize and potato crops had a greater impact on NTAs compared with *Bt* technologies.

In summary, results from the present multi-site study suggest that a *Bt* cotton technology expressing transgenic events DAS-21023-5 × DAS-24236-5 × SYN-IR102-7 will be an important tool that offers high and expanded efficacy to control target key and secondary lepidopteran pests affecting a cotton crop without adverse effects on the NTA community associated with cotton fields. Results presented herein document the first detailed report for the susceptibility of *S*. *frugiperda*, *S*. *cosmioides*, *C*. *includens*, *C*. *virescens* and *A*. *argillacea* larvae to *Bt* cotton expressing Cyr1F + Cry1Ac + Vip3Aa19 proteins associated with transgenic events DAS-21023-5 × DAS-24236-5 × SYN-IR102-7. In addition, this is the first effort to assess the impact of this *Bt* technology on the NTA community associated with a cotton crop in commercial cotton areas of Brazil.

## Supporting information

S1 Table Gradient lengths (SD units) via Detrended Correspondence Analysis (DCA) of the most representative non-target arthropods (NTAs) collected in *Bt* cotton technology expressing the events DAS-21023-5 × DAS-24236-5 × SYN-IR102-7 and non-*Bt* cotton plots at three sites in Brazil (2014/2015 cropping season). (DOCX)

S2 Table Two-way repeated-measures ANOVA results (α = 0.05) for abundance of non-target arthropods (NTAs) that contributed most to the community response in the PRC analysis (weights greater than 0.5 or less than -0.5) with first axis significant. The NTAs were collected in *Bt* cotton technology expressing the events DAS-21023-5 × DAS-24236-5 × SYN-IR102-7 and non-*Bt* cotton plots in Brazil (2014/2015 cropping season).
(DOCX)
