# Supplementary material for: Field efficacy of Bt cotton containing events DAS-21023-5 × DAS-24236-5 × SYN-IR102-7 against lepidopteran pests and impact on the non-target arthropod community in Brazil
Source: PLoS One. 2021 May 4;16(5):e0251134. doi: 10.1371/journal.pone.0251134 (PMC8096009; doi:10.1371/journal.pone.0251134)
Supplement: S1 Table — (DOCX) [file pone.0251134.s001.docx]

**S1 Table.** Gradient lengths (SD units) via Detrended Correspondence Analysis (DCA) of the most representative non-target arthropods (NTAs) collected in *Bt* cotton technology expressing the events DAS-21023-5 × DAS-24236-5 × SYN-IR102-7 and non-*Bt* cotton plots at three sites in Brazil (2014/2015 cropping season).

| Sampling methods | Sites | | |
| --- | --- | --- | --- |
|  | Conchal | Indianópolis | Montividiu |
| *Aerial-dwelling NTAs* |  |  |  |
| Beat cloth | 3.0 | 3.3 | 2.8 |
| Sticky traps | 1.7 | 2.1 | 2.1 |
| *Ground-dwelling NTAs* |  |  |  |
| Pitfall traps | 3.4 | 2.4 | 2.2 |
| Berlese-Tullgren funnel | 2.8 | 3.2 | 1.4 |

Gradient lengths shorter than 4.0 indicate appropriate application of linear response models in redundancy analysis (Lepš and Šmilauer, 2003).
