# Supplementary material for: Field efficacy of Bt cotton containing events DAS-21023-5 × DAS-24236-5 × SYN-IR102-7 against lepidopteran pests and impact on the non-target arthropod community in Brazil
Source: PLoS One. 2021 May 4;16(5):e0251134. doi: 10.1371/journal.pone.0251134 (PMC8096009; doi:10.1371/journal.pone.0251134)
Supplement: S2 Table — The NTAs were collected in Bt cotton technology expressing the events DAS-21023-5 × DAS-24236-5 × SYN-IR102-7 and non-Bt cotton plots in Brazil (2014/2015 cropping season). (DOCX) [file pone.0251134.s002.docx]

**S2 Table.** Two-way repeated-measures ANOVA results (α = 0.05) for abundance of non-target arthropods (NTAs) that contributed most to the community response in the PRC analysis (weights greater than 0.5 or less than -0.5) with first axis significant. The NTAs were collected in *Bt* cotton technology expressing the events DAS-21023-5 × DAS-24236-5 × SYN-IR102-7 and non-*Bt* cotton plots in Brazil (2014/2015 cropping season).

| Sampling method | Site | Non-target arthropod | Two-way RM-ANOVA | | | | | |
| --- | --- | --- | --- | --- | --- | --- | --- | --- |
|  |  |  | Cotton technologies (A) | | Sampling time (B) | | Interaction (A x B) | |
|  |  |  | F | *P* | F | *P* | F | *P* |
| Beat cloth | Conchal | Aphididae sp. | 4.8 | 0.117 | 13.4 | < 0.001 | 13.4 | < 0.001 |
|  |  | Araneae sp. | 0.7 | 0.457 | 23.4 | < 0.001 | 3.9 | 0.018 |
|  |  | *Doru luteipes* | 5.5 | 0.101 | 58.3 | < 0.001 | 1.0 | 0.427 |
|  |  | *Dorymyrmex brunneus* | 0.9 | 0.417 | 16.2 | < 0.001 | 0.9 | 0.526 |
|  |  | Formicidae sp. | 0.4 | 0.562 | 9.1 | < 0.001 | 0.3 | 0.936 |
|  |  | *Hippodamia convergens* | 3.0 | 0.182 | 3.9 | 0.017 | 2.6 | 0.072 |
|  |  | *Lagria villosa* | 19.1 | 0.022 | 3.3 | 0.032 | 0.6 | 0.675 |
|  |  | *Orius* sp. | 10.7 | 0.047 | 17.2 | < 0.001 | 5.8 | 0.004 |
|  |  | Thysanoptera sp. | 0.0 | 0.970 | 21.6 | < 0.001 | 3.5 | 0.026 |
|  |  |  |  |  |  |  |  |  |
| Sticky traps | Indianópolis | Agromyzidae sp. | 0.0 | 0.889 | 62.9 | < 0.001 | 1.6 | 0.215 |
|  |  | Aphididae sp. | 0.2 | 0.721 | 254.8 | < 0.001 | 1.8 | 0.180 |
|  |  | Apoidea sp. 03 | 4.6 | 0.121 | 0.9 | 0.482 | 0.6 | 0.731 |
|  |  | Bethylidae sp. 01 | 5.9 | 0.096 | 98.9 | < 0.001 | 7.1 | 0.001 |
|  |  | *Caliothrips* sp. | 0.5 | 0.533 | 0.7 | 0.662 | 1.7 | 0.199 |
|  |  | *Chelonus* sp. | 0.1 | 0.824 | 3.1 | 0.040 | 2.8 | 0.055 |
|  |  | Chloropidae sp. 02 | 0.4 | 0.555 | 72.9 | < 0.001 | 1.7 | 0.194 |
|  |  | Cicadellidae sp. | 0.5 | 0.546 | 21.4 | < 0.001 | 1.8 | 0.181 |
|  |  | *Coenosia* sp. | 2.9 | 0.188 | 18.3 | < 0.001 | 1.1 | 0.411 |
|  |  | *Cycloneda sanguinea* | 1.4 | 0.325 | 107.6 | < 0.001 | 0.2 | 0.939 |
|  |  | *Diabrotica speciosa* | 0.6 | 0.484 | 14.0 | < 0.001 | 1.9 | 0.139 |
|  |  | Diptera 11 | 0.1 | 0.751 | 49.3 | < 0.001 | 0.8 | 0.573 |
|  |  | Diptera 16 | 0.8 | 0.434 | 18.1 | < 0.001 | 7.8 | < 0.001 |
|  |  | *Elachiptera* sp. | 1.2 | 0.346 | 11.8 | < 0.001 | 2.8 | 0.058 |
|  |  | Empididae sp. | 2.7 | 0.198 | 3.6 | 0.024 | 1.6 | 0.223 |
|  |  | Eucoilinae sp. 02 | 2.5 | 0.212 | 8.8 | < 0.001 | 4.0 | 0.016 |
|  |  | *Frankliniella occidentalis* | 1.3 | 0.331 | 150.1 | < 0.001 | 3.2 | 0.037 |
|  |  | *Frankliniella schultzei* | 2.3 | 0.230 | 97.4 | < 0.001 | 3.2 | 0.038 |
|  |  | *Hippodamia convergens* | 0.8 | 0.441 | 5.7 | 0.004 | 0.8 | 0.576 |
|  |  | Hymenoptera 13 | 0.2 | 0.698 | 3.3 | 0.033 | 2.8 | 0.057 |
|  |  | Ichneumonidae sp. 29 | 14.1 | 0.033 | 6.3 | 0.003 | 8.6 | < 0.001 |
|  |  | *Lysiphlebus testaceipes* | 0.2 | 0.717 | 2.9 | 0.050 | 1.2 | 0.366 |
|  |  | *Megaselia scalaris* | 0.5 | 0.519 | 47.5 | < 0.001 | 0.8 | 0.536 |
|  |  | Pipunculidae sp. 02 | 0.2 | 0.660 | 3.3 | 0.034 | 1.7 | 0.193 |
|  |  | Platygastridae sp. 02 | 13.8 | 0.034 | 4.9 | 0.008 | 2.0 | 0.137 |
|  |  | Sarcophagidae sp. | 1.7 | 0.284 | 53.2 | < 0.001 | 2.2 | 0.113 |
|  |  | Staphylinidae sp. | 0.9 | 0.393 | 22.9 | < 0.001 | 2.2 | 0.115 |
|  |  | *Stelidota* sp. | 6.2 | 0.088 | 35.7 | < 0.001 | 2.9 | 0.057 |
|  |  | Tachinidae sp. | 3.7 | 0.149 | 38.1 | < 0.001 | 1.4 | 0.274 |
|  |  | Therevidae sp. 01 | 1.3 | 0.336 | 9.0 | < 0.001 | 1.2 | 0.353 |
|  |  |  |  |  |  |  |  |  |
| Sticky traps | Montividiu | *Coenosia* sp. | 0.0 | 0.907 | 2.5 | 0.078 | 12.7 | < 0.001 |
|  |  | Diapriidae sp. 03 | 3.7 | 0.149 | 11.8 | < 0.001 | 2.3 | 0.096 |
|  |  | Diptera 49 | 2.3 | 0.228 | 19.7 | < 0.001 | 12.4 | < 0.001 |
|  |  | Drosophilidae sp. | 1.1 | 0.367 | 27.9 | < 0.001 | 16.8 | < 0.001 |
|  |  | Eucoilinae sp. 02 | 38.6 | 0.008 | 119.3 | < 0.001 | 44.9 | < 0.001 |
|  |  | *Euxesta* sp. | 0.4 | 0.593 | 23.1 | < 0.001 | 4.7 | 0.009 |
|  |  | Phoridae sp. | 10.8 | 0.046 | 54.5 | < 0.001 | 65.8 | < 0.001 |
|  |  | Sarcophagidae sp. | 0.2 | 0.662 | 19.8 | < 0.001 | 2.5 | 0.075 |
|  |  | Sciaridae sp. | 0.0 | 0.936 | 13.5 | < 0.001 | 10.8 | < 0.001 |
|  |  | Thysanoptera sp. 02 | 0.8 | 0.435 | 3.7 | 0.022 | 5.4 | 0.005 |
|  |  | Thysanoptera sp. 03 | 9.3 | 0.055 | 29.8 | < 0.001 | 5.8 | 0.003 |
|  |  |  |  |  |  |  |  |  |
| Pitfall traps | Montividiu | *Calosoma* sp. | 85.1 | 0.003 | 8.7 | < 0.001 | 9.8 | < 0.001 |
|  |  | *Canthon* sp. 02 | 1.5 | 0.302 | 3.3 | 0.033 | 1.4 | 0.282 |
|  |  | Dermaptera (nymph) | 8.1 | 0.066 | 7.8 | < 0.001 | 7.8 | < 0.001 |
|  |  | *Dorymyrmex brunneus* | 6.7 | 0.082 | 4.1 | 0.016 | 8.2 | < 0.001 |
|  |  | *Galerita* sp. | 2.3 | 0.227 | 10.8 | < 0.001 | 26.3 | < 0.001 |
|  |  | *Gryllus assimilis* | 0.2 | 0.709 | 22.7 | < 0.001 | 1.5 | 0.248 |
|  |  | *Labidura xanthopus* | 3.7 | 0.150 | 9.4 | < 0.001 | 1.5 | 0.243 |
|  |  | Orthoptera (nymph) | 4.8 | 0.117 | 25.7 | < 0.001 | 6.8 | 0.002 |

Degrees of freedom: cotton technologies = 1; sampling time = 5; interaction = 5; residual = 15.
